# Flux Design: In silico design of cell factories based on correlation of pathway fluxes to desired properties

**DOI:** 10.1186/1752-0509-3-120

**Published:** 2009-12-25

**Authors:** Guido Melzer, Manely Eslahpazir Esfandabadi, Ezequiel Franco-Lara, Christoph Wittmann

**Affiliations:** 1 Institute of Biochemical Engineering, Technische Universität Braunschweig, Gaußstr 17, 38106 Braunschweig, Germany

## Abstract

**Background:**

The identification of genetic target genes is a key step for rational engineering of production strains towards bio-based chemicals, fuels or therapeutics. This is often a difficult task, because superior production performance typically requires a combination of multiple targets, whereby the complex metabolic networks complicate straightforward identification. Recent attempts towards target prediction mainly focus on the prediction of gene deletion targets and therefore can cover only a part of genetic modifications proven valuable in metabolic engineering. Efficient in silico methods for simultaneous genome-scale identification of targets to be amplified or deleted are still lacking.

**Results:**

Here we propose the identification of targets via flux correlation to a chosen objective flux as approach towards improved biotechnological production strains with optimally designed fluxes. The approach, we name Flux Design, computes elementary modes and, by search through the modes, identifies targets to be amplified (positive correlation) or down-regulated (negative correlation). Supported by statistical evaluation, a target potential is attributed to the identified reactions in a quantitative manner. Based on systems-wide models of the industrial microorganisms *Corynebacterium glutamicum *and *Aspergillus niger*, up to more than 20,000 modes were obtained for each case, differing strongly in production performance and intracellular fluxes. For lysine production in *C. glutamicum *the identified targets nicely matched with reported successful metabolic engineering strategies. In addition, simulations revealed insights, e.g. into the flexibility of energy metabolism. For enzyme production in *A.niger *flux correlation analysis suggested a number of targets, including non-obvious ones. Hereby, the relevance of most targets depended on the metabolic state of the cell and also on the carbon source.

**Conclusions:**

Objective flux correlation analysis provided a detailed insight into the metabolic networks of industrially relevant prokaryotic and eukaryotic microorganisms. It was shown that capacity, pathway usage, and relevant genetic targets for optimal production partly depend on the network structure and the metabolic state of the cell which should be considered in future metabolic engineering strategies. The presented strategy can be generally used to identify priority sorted amplification and deletion targets for metabolic engineering purposes under various conditions and thus displays a useful strategy to be incorporated into efficient strain and bioprocess optimization.

## Background

The identification of genetic target genes is a key step in rational engineering of production strains towards bio-based chemicals, fuels or therapeutics. To fully account for the high complexity of metabolic networks and select promising genes out of many possible candidates, systems-wide approaches have recently emerged from the rapidly increasing amount of genome-scale models [1]. As example, OptKnock [2] OptGene [3], minimization of metabolic adjustment (MOMA) [4] as well as strain design based on optimum theoretical yield [5] display efficient in *silico *algorithms that allow the prediction of promising gene deletion targets towards overproduction of chemicals. They do, however, not provide a prediction of genes to be amplified for superior performance. This rather important information on potential amplification targets can be extracted on basis of experimental ^13^C metabolic flux data including comparative ^13^C flux studies of mutants with different properties [6] or a bi-level optimization framework (OptReg) which predicts gene amplification, attenuation or deletion targets on the basis of experimental flux data and regulation strength parameters [7]. The value of such approaches, exploiting ^13^C flux data, has been successfully demonstrated e. g. for lysine producing *C. glutamicum *[8,9]. They, however, require the availability of experimental data as basis of identifying amplification targets which is linked to increased experimental effort and might not give access to all potentially interesting gene candidates. Also metabolic control analysis, allowing the prediction of rate-limiting steps, gives access to amplification targets, but relies on experimentally data, e.g. *in vivo *kinetic data of the enzymes involved [10]. Thus, efficient in silico methods for simultaneous genome-scale identification of targets to be amplified or deleted, which do not rely on available experimental data or a priori assumptions, are still lacking.

Among the available genome-scale modelling approaches, elementary flux mode analysis constitutes an important tool for the efficient study of cellular systems, since it allows the *in silico *prediction of desirable cell phenotypes that result either from the variation of process parameters or from the perturbation of genotypes [11]. In comparison to alternative methods, such as linear programming, elementary flux mode analysis enables the investigation of all possible physiological states in the cell and can identify all existing metabolic flux vectors without any a priori knowledge or assumption on measured fluxes [5]. Elementary flux mode analysis has been applied to predict promising gene deletion strategies as shown for rational design of L-methionine production in bacteria [12], the identification of genetically independent pathways in recombinant yeast [13] or the construction of a minimal *E. coli *cell for high yield ethanol production was enabled by prediction of gene deletion targets using elementary flux mode analysis [5]. Here we present an in silico approach for quantitative target prediction towards superior cell factories. To this end we extend elementary flux mode analysis to a network-wide search for flux changes among all possible modes which are specifically correlated to a chosen target flux, i.e. the production capacity of the cell. Recent modelling studies showed that such a coupling of fluxes is an important behaviour of biological systems e.g. with respect to co-regulation of genes [14]. However, a direct application towards target identification and superior production strains has not been considered. The potential of our approach is demonstrated for industrially relevant cell factories of different complexity. The soil bacterium *C. glutamicum *is one of the dominating bacteria in biotechnology and applied to produce more than 2.000.000 tons of amino acids per year [15]. Its valuable product lysine, almost exclusively derived through fermentation by this microorganism, is used in animal nutrition. Due to its high relevance, *C. glutamicum *has been extensively investigated including the construction of a genome-scale model [16] and different success stories towards optimization lysine production by metabolic engineering which display an excellent basis as relevant test case for the simulations shown here [17]. The filamentous fungus *A. niger *is widely exploited for the production higher-value enzyme products [18]. The recently published genome-wide network model of *A. niger *illustrates its complex metabolism located in different intracellular compartments [19]. Here we focus on the industrial enzymes fructofuranosidase, used to obtain valuable oligosaccharides [20], glucoamylase, applied in starch conversion [21], and epoxide hydrolyase, a highly useful biocatalyst for kinetic resolution of racemic epoxides [22].

## Methods

### Computation of elementary flux modes

Computation of elementary flux modes allows the calculation of a solution space of all possible independent metabolic pathways in a steady-state [23]. Elementary flux modes are thermodynamically and stoichiometrically possible pathways reducing the complex metabolism into all, unique, non-decomposable biochemical pathways [24], which connect the supplied substrates with the corresponding end products. Algorithms for computing elementary flux modes are based on two fundamental equations. Assuming the existence of a (quasi) steady-state metabolism throughout the metabolic network, the first fundamental balancing equation can be written as:(1)

Here, the metabolic network is expressed as stoichiometric matrix **S **with the dimension dim **S **= (***m ***× ***q***), where ***m ***is the number of internal metabolites and ***q ***is the number of reactions, and **r **represents a flux distribution and is consequently a ***q ***× 1 vector. Any biochemical reaction network should fulfil the thermodynamic feasibility constraint, i.e. the following inequality should be valid for all irreversible reaction rates:(2)

In the present work, elementary flux mode calculation was performed using the double description method (null space approach) introduced by Wagner [25] and extended with the recursive enumeration strategy with bit pattern trees by Terzer and Stelling [26]. An implementation of the algorithm in Java, with integration into MatLab (Mathworks Inc., Natick, MA) is available at http://csb.inf.ethz.ch and was applied in this work. On basis of the determined elementary modes, a detailed investigation of metabolic network properties was carried out. This included the estimation of theoretical (maximum) yield, relative fluxes through intracellular metabolic pathways, and target prediction for strain engineering. Calculations were partially automated and implemented into MatLab (Mathworks Inc., Natick, MA) and evaluated in Excel™ (Microsoft Office 2007, version 12.0).

### Calculation of relative flux normalized to substrate uptake

For a given elementary mode *j *the relative metabolic flux (ν_*i*, *j*_) of each metabolic reaction *i*, normalized to the substrate uptake flux, was determined. The symbol ξ refers to the molar carbon content expressed in c-mol per mol. To facilitate direct comparison between different carbon sources, all relative fluxes were normalized to one unit of hexose (Eqs. 3, 4). The variable ***q ***refers to the number of metabolic reactions in the metabolic network. The variable **n **refers to the numbers of elementary modes respectively.(3)

### Calculation of theoretical (maximum) yield

The theoretical product (Y_P/C, *j*_) and biomass yield (Y_X/C, *j*_) was calculated for each elementary mode *j *according to Eq. 5. Basically it displays the relative flux towards the product or the biomass. The variable **s **refers to the stoichiometric coefficient of the product (***P***) and carbon source (***C***)(5)

Since every real flux distribution in a biological system is a linear combination of elementary modes, the mode with the highest product or biomass yield, respectively, gives direct access to the maximum capacity of the underlying network, i.e. the maximum theoretical yields Y_P/C, *max*_, and Y_X/C, *max*_.

### Target potential based on flux correlation

To investigate, whether a reaction *i *displays a potential target, a chosen set of elementary flux modes was searched for statistically relevant correlation between the relative flux through the objective reaction *obj *and that through the reaction *i*. For this purpose the slope of the linear regression between the objective flux (ν_*obj*_) and the corresponding flux (ν_*i*_) was determined. This was carried out for each reaction, so that the entire network could be screened for potential targets. Only statistically valid correlations were considered further. For this purpose a cut-off value of r^2 ^= 0.7 was set for the regression coefficient of each linear correlation. Such a cut-off has proven valid in previous studies processing correlated data [27,28]. Additionally, the statistical significance of these targets was further proven by the t-test (Eq. 6).(6)

Here, the variable n is the number of pairs of values, r is the correlation coefficient and r^2 ^the regression coefficient. If, TS > t(f, P), then there exists a statistically significant relationship. Accordingly, statistical significance was a quality criterion to classify the corresponding reaction as a potential target. Subsequently, the potential of a metabolic reaction as genetic target was expressed as target potential coefficient (α_i, obj_), by the slope of the corresponding linear regression α_i, obj _= (ν_i _± β_i, obj_)/ν_*obj*_, whereby β_i, obj _is the intercept of the ordinate (Eq. 7).(7)

The calculation was carried out by determining the covariance (cov) of the variables of ν_*obj *_and ν_i _divided by the square of the standard deviation ***δ ***of the corresponding objective flux ν_*obj *_(Eq. 8).(8)

Positive values of α_i, obj _account for amplification targets, whereas negative values denote deletion or attenuation targets.

## Metabolic modelling

The major characteristics of the models used in the present work were as follows. A detailed description of the biochemical reactions in the different networks is given in the supplement files.

### Small example network of TCA cycle and supporting pathways

The principle of the developed approach is elucidated using a simple metabolic network from *E. coli*, which was previously used for the discussion of the concept of elementary flux mode analysis [24]. It includes the TCA cycle, the glyoxylate shunt and connected reaction of amino acid bio-synthesis. In this example, 2-phosphoglycerate, ammonium, carbon dioxide, and the cofactors, such as ATP and NAD, are considered as external metabolites. Arbitrarily, succinyl-CoA was defined as desired product and its formation as objective reaction. The stoichiometric equations of the metabolic model are listed in the supplement [Additional file 1].

### Metabolic network of *C. glutamicum*

The metabolic reaction model of *C. glutamicum *considered the actual knowledge from the genome scale model recently created [16]. It included all relevant pathways of central carbon, nitrogen and sulphur metabolism as well as the entire subset of anabolism and the corresponding reactions linked to formation and secretion of extracellular products. For elementary flux mode analysis, 7 external compounds were considered including the substrates glucose, ammonium, sulphate and oxygen and the products lysine, biomass and carbon dioxide. Additionally, ATP, required for maintenance, was considered as an external metabolite. The stoichiometric equation for biomass synthesis included all relevant precursor metabolites. The relative amount and composition of the macromolecules DNA, carbohydrates, lipids, protein and RNA was taken from thorough analysis of cellular composition [29]. For ATP production from NADH and menaquinol in the respiratory chain, a P/O ratio of 2 was assumed [16]. The stoichiometric equations of the metabolic model are listed in the supplement [Additional file 2].

### Metabolic network of *Aspergillus niger*

The metabolic reaction model of the central metabolism of *A. niger *contained was constructed on basis of the genome scale model recently published [19]. The model included all relevant pathways of central carbon, nitrogen and sulphur metabolism as well as the entire subset of anabolism and the corresponding reactions linked to formation of extracellular products. Hereby, the cellular compartment mitochondrion, glyoxysome and cytosol were considered together with the respective transport reactions. For elementary flux mode analysis, external compounds were substrates (sources of carbon, nitrogen, sulphur, oxygen) and products (enzyme, biomass, carbon dioxide, gluconate, oxalate, citrate). Additionally, ATP for maintenance was included in the model and considered as an external metabolite. For ATP production the P/O ratio for mitochondrial NADH was assumed as 2.64 and that for succinate and cytosolic NADH as 1.64 [19]. The stoichiometric equation for biomass synthesis included all relevant precursor metabolites from the central carbon metabolism. The relative amount and composition of the macromolecules DNA, glucan, glycogen, lipid and RNA was taken from [30]. The amino acid composition of the cell protein was calculated from the average protein content of *A.niger *using the program IdentiCS [31]. Glycosylation of cellular protein was considered, taking Galf_2_Man_8_(GlcNAc) as average composition of the glycosylation residues in filamentous fungi [32] and an average number of 33 sugar residues [33] into account. This resulted in the stoichiometric fraction of Galf_6_Man_24_(GlcNAc)_3 _per protein. For the calculation of the exact demand it was assumed that on average 64% of all proteins are glycosylated [34]. The cellular demand for synthesis of the enzymes fructofuranosidase, glucoamylase and epoxide hydrolase was calculated as follows. Fructofuranosidase is highly glycosylated [20], whereby half of the enzyme consists of glycosylation chains (NetNGlyc, http://www.cbs.dtu.dk/). Hereby, the glycosylation pattern Galf_18_Man_308_(GlcNAc)_8.5, _as previously determined for this enzyme, was considered [35]. The amino acid composition of fructofuranosidase was derived from the corresponding open reading frame-ID An08 g11070 [36]. Similarly, the amino acid composition (An03 g06550) and the glycosylation pattern [37] was taken into account for glucoamylase. Epoxide hydrolase is non-glycosylated so that only the protein itself had to be considered (An16 g02170). The stoichiometric equations of the metabolic model are listed in the supplement [Additional file 3].

## Results

### Target identification based on flux correlation - small example network

**Figure 1:**
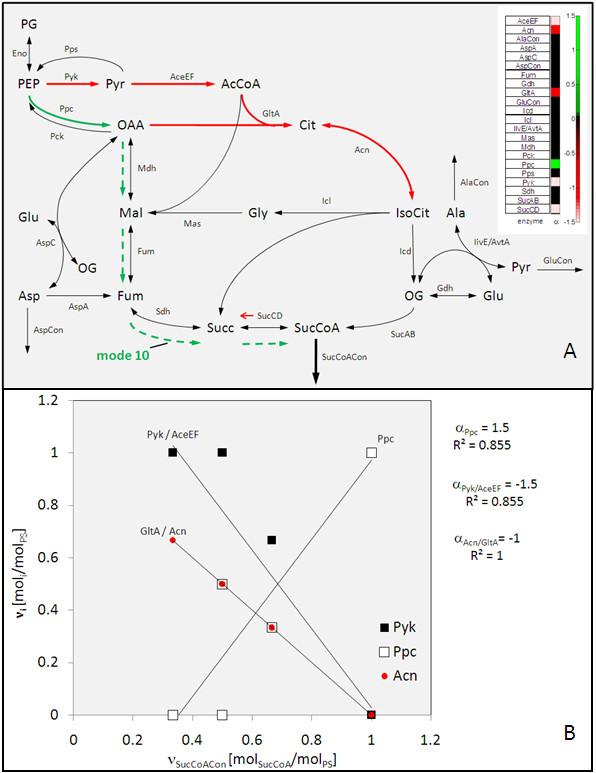
**Principle of target identification by search for flux correlation to desired properties, here succinyl-CoA production in a small example network taken from **[24]. Calculation (A) of the target potential α by correlation analysis and data visualization (B) as heat map or in network form with colour coded representation of amplification targets (solid green arrow) and deletion targets (red arrows).

**Figure 2:**
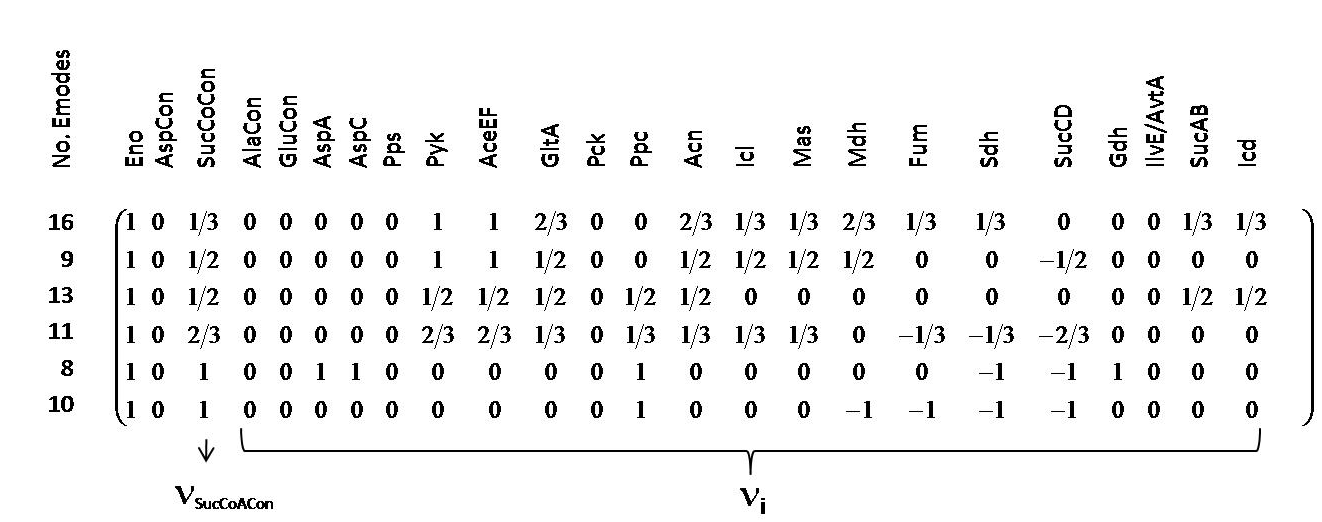
**Stoichiometric matrix including all succinyl-CoA producing elementary modes which are normalized to the substrate uptake reaction (Eno) in the first column**. The modes are sorted with increasing size of the succinyl-CoA yield ν_SucCoACon _(reaction: SucCoACon).

A small network comprising TCA cycle, glyoxylate shunt and connected amino acid metabolism from *E. coli *serves as example to introduce the principle of the developed approach for target identification (Figure 1A). In the present example, succinyl-CoA is considered as desired product. The network comprises 16 different elementary modes (see also [24]). These display the basic solution space for the prediction of amplification and deletion targets. In a first step, all flux modes with zero flux towards the target product are eliminated, resulting in a subset of 6 relevant modes. Subsequently, the remaining modes are normalized to the substrate entry reaction (here enolase) and arranged in matrix form (Figure 2). Obviously, the modes differ in the objective flux which is linked to substantial differences in the other network fluxes. This can now be exploited by scanning through the network reactions for their correlation to the objective flux as exemplified in Figure 1B. Several reactions show insignificant or even no correlation. Phosphoenolpyruvate carboxylase (Ppc), however, is clearly identified as amplification target. Moreover, a number of reactions, including pyruvate kinase (Pyk), pyruvate dehydrogenase (aceEF), citrate synthase (GltA), aconitase (Can) and succinyl-CoA dehydrogenase (SucCD) reveal negative correlation, i. e. are identified as deletion or attenuation targets. The visualization of the resulting target potential coefficient (α) as heat map or in network form provides direct access to promising targets with ranked priority (Figure 1A).

### Lysine production in *C. glutamicum*

#### Maximum production performance using glucose as carbon source

**Figure 3:**
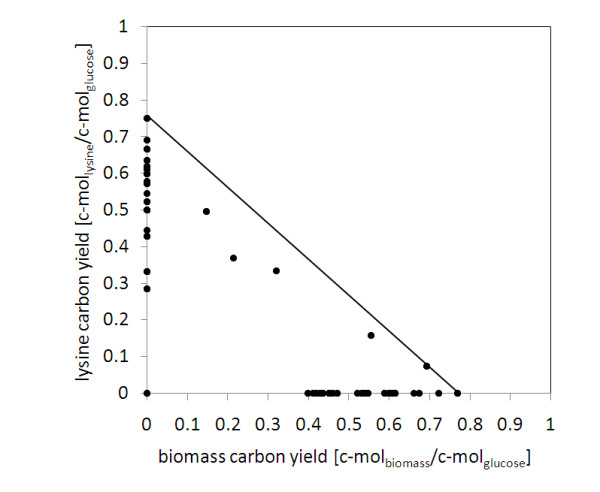
**Elementary modes for lysine and biomass production in *C. glutamicum *on glucose the solution space of the elementary modes, represented by the black dots, is marked through the interior as well as the sides of the rectangular triangle**. The modes on the axes represent extreme modes exclusively linked to production of lysine or biomass.

**Figure 4:**
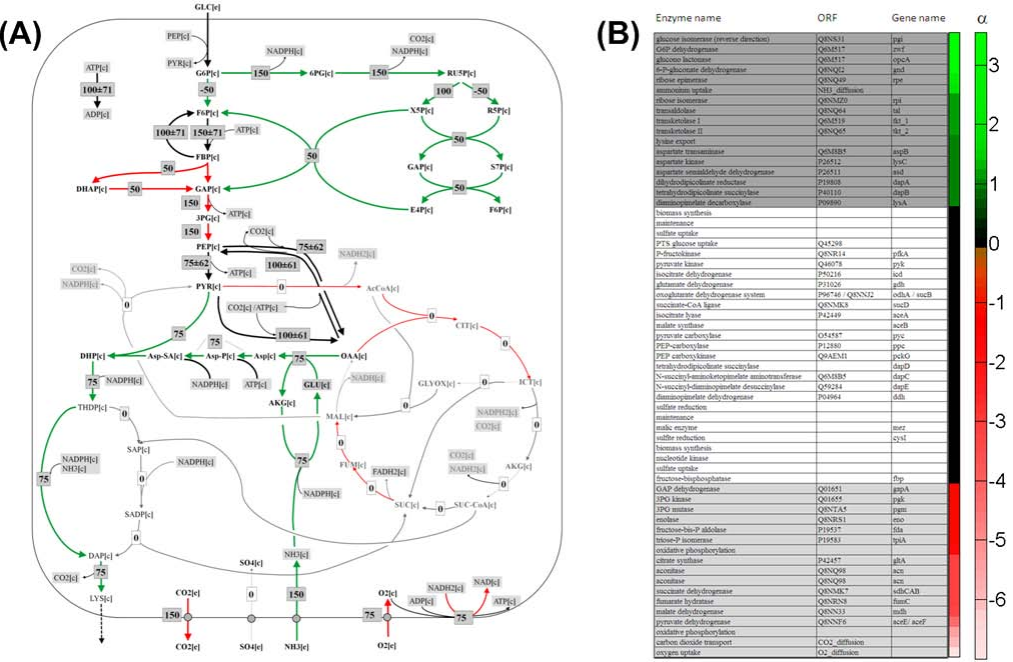
**Prediction of genetic targets for improved lysine production in *C. glutamicum *based on correlation of flux through metabolic reactions with lysine production flux among the calculated elementary modes: Optimal flux distribution for lysine production in *Corynebacterium glutamicum *on glucose as obtained from elementary mode analysis (A) and resulting target potential coefficients (B)**. In the flux map all fluxes are given as relative molar flux normalized to the uptake flux. The data shown display the average fluxes and deviations from the different elementary modes under optimum production conditions. The coloured arrows reflect amplification (green) and deletion/attenuation targets (red). In the heat map, listing the predicted targets, a positive value (green) relates to a reaction, which positively correlates with the production (amplification target), whereas negative correlation (red) displays a deletion/attenuation target. Black colour indicates statistically insignificant values.

Overall, 289 modes resulted for lysine production in *C. glutamicum*. As shown, a large number of elementary flux modes with different yield for lysine and biomass were obtained (Figure 3). Among the modes observed, the majority are extreme modes exclusively linked to production of either biomass or lysine. These are given on the two axes of the plot. In addition also flux modes with simultaneous production of biomass and lysine resulted. Among all modes, 6 modes enabled the optimum yield of 0.75 (mol lysine)/(mol glucose) which agrees with the value obtained by flux balance analysis [16]. The average flux map from these optimum modes reveals the key pathways contributing to efficient lysine formation such as pentose phosphate pathway, ammonium metabolism, lysine biosynthesis and secretion (Figure 4A). The flux through most of these pathways is conserved. ATP linked reactions, however, reveal a substantial flexibility. The consumption of ATP under optimum production conditions either involves cellular maintenance requirement or "futile" cycling recruiting the carboxylation and decarboxylation reactions at the pyruvate node or the two enzymes phosphofructokinase and fructose bisphosphatase.

#### Prediction of amplification and deletion targets

The obtained alternative optima and the various interesting suboptimal solutions now provided a rich source for target search. The elementary modes were now screened for statistically significant correlation of fluxes as indicator of targets to be amplified or deleted. Most targets were identified for the subset of non-growth modes which do not exhibit biomass formation. Here, flux correlation analysis clearly identified a number of reactions as potential targets (Figure 4B). Targets to be amplified are attributed to all reactions of the pentose phosphate pathway, as well as ammonium uptake and assimilation, different enzyme of the lysine biosynthesis and the lysine secretion. Interestingly, also the entry enzyme into the glycolysis, glucose 6-phosphate isomerase is classified as amplification target. This can be understood from its role in re-cycling carbon back into the pentose phosphate cycle enabled by its reversible nature (Figure 4A). Deletion or attenuation targets are located in the glycolysis, the TCA cycle and also the oxidative respiratory system. When ranked by priority, i.e. the value of the target potential coefficient α, the most striking targets predicted are located at the glucose 6-phosphate node, which reveal this node as key to successful engineering of *C. glutamicum *for improved lysine production. The simultaneous consideration of the potential targets reveals a systems-wide redirection of flux towards a superior producer as indicated by the desired flux distribution at optimal performance (Figure 4A).

### Enzyme production in *Aspergillus niger*

#### Maximum production performance using glucose as carbon source

**Figure 5:**
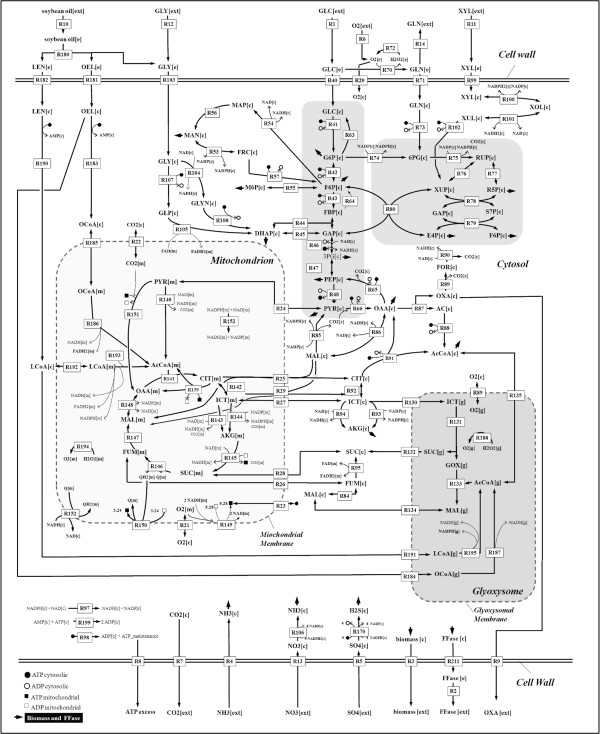
**Metabolic model for *Aspergillus niger***. Reactions and metabolites are compartmentalized between extracellular [e], cytosolic [c], mitochondrial [m] and glyoxysomal [g] compartments. Numbers next to the arrows refer to the detailed model description in the supplement.

**Figure 6:**
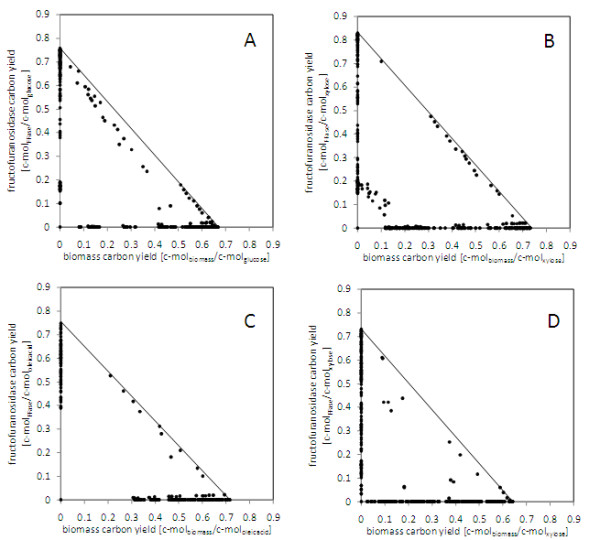
**Comparison of elementary modes for biomass and fructofuranosidase production in *A. niger *on different carbon sources**. A: glucose, B: glycerol, C: soybean oil, D: xylose. The solution space of the elementary modes, represented by the black dots, is marked through the interior as well as the sides of the rectangular triangle. The modes on the axes represent extreme modes exclusively linked to production of biomass or fructofuranosidase (FFase).

Figure 5 presents a condensed view of the metabolic network of *A.niger *for the production of fructofuranosidase. Overall, about 21.100 modes were obtained on glucose and ammonium. The modes differed substantially in the corresponding yield for the enzyme or the biomass (Figure 6A). The dominating fraction of modes was linked to exclusive production of either fructofuranosidase or biomass, respectively. The maximal carbon yield was 0.76 c-mol/c-mol for fructofuranosidase and 0.67 c-mol/c-mol for biomass (Table 1). In comparison, 1986 elementary modes (9%), located within the interior of the triangular solution space, exhibited simultaneous formation of both compounds. Only 0.8% of all modes allowed maximum enzyme yield, all at zero growth.

**Table 1:** Elementary flux mode analysis of fructofuranosidase (FFase) production by *A. niger *on different carbon and nitrogen sources.

	Maximum carbon yield [c-mol/c-mol]			Number of Elementary Modes	
	
Carbon Source/Nitrogen Source	FFase	Biomass	Total	Modes linked to FFase production (% of total EFM)	Modes linked to biomass and FFase production (% of total EFM)
glucose/NH3	0.76	0.67	21,147	7,045 (33)	1,986 (9)
glycerol/NH3	0.83	0.73	21,122	8,070 (38)	2,267 (11)
oleic acid/NH3	0.75	0.72	20,895	9,071 (43)	1,702 (8)
xylose/NH3	0.73	0.64	13,364	3,896 (29)	187 (1)
glucose/NO3	0.61	0.54	29,435	13,160 (45)	1,425 (5)
glycerol/NO3	0.67	0.59	33,462	14,090 (42)	2,984 (9)
oleic acid/NO3	0.65	0.59	27,753	12,652 (46)	1,724 (6)
xylose/NO3	0.59	0.52	24,098	8,443 (35)	249 (5)

#### Optimal pathways for glucose based production

**Figure 7:**
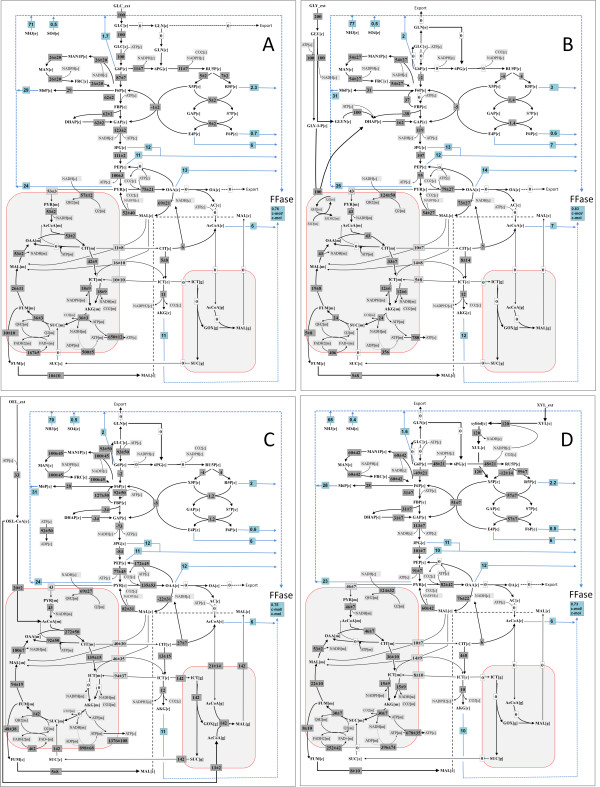
**Optimal flux distribution for fructofuranosidase production in *A. niger***. A: glucose, B: glycerol, C: soybean oil, D: xylose. The relative fluxes are averaged from 52 (glucose), 89 (glycerol), 354 (soybean oil) and 48 (xylose) elementary flux modes for maximal fructofuranosidase production obtained. All fluxes are given as relative molar flux normalized to 1 mol of hexose unit [mol. (mol hexose)^-1^.100].

The average flux distribution from the modes with maximum enzyme yield provides a detailed picture on the reactions involved (Figure 7A). The contribution the non-oxidative PPP, the glycolysis, the fructofuranosidase synthesis as well as transport processes was rather constant as indicated by the low deviation of corresponding fluxes. Other reactions showed a higher flexibility suggesting that key functions of the network under optimum production conditions can be realized by different flux states. Interestingly, this included a number of cytosolic enzymes which are all involved in supply of NADPH, i.e. the oxidative PPP, malic enzyme and isocitrate dehydrogenase as well as mannitol 2-phosphate dehydrogenase. Furthermore, maximum production was linked to zero by-product formation. The entire ATP formed was completely recruited for fructofuranosidase production.

#### Impact of alternative carbon and nitrogen source

Elementary flux mode analysis was further carried out for the industrially relevant carbon sources xylose, glycerol and oleic acid (Table 1). The reduced substrate glycerol revealed an optimal production of 0.83 c-mol/c-mol and was the best carbon source (Figure 6B). Oleic acid (0.72 c-mol/mol) and xylose (0.73 c-mol/c-mol) were slightly less efficient Figures 6C, D). Glycerol was metabolized by simultaneous usage of the NADH-dependent glycerol-dehydrogenase and the FAD-dependent glycerol 3-phosphate dehydrogenase (Figure 7B). Due to this reducing equivalents were released into the cytosol and mitochondrion, respectively. This caused an increased flux through the NADH-ubiquinone oxidoreductase, counterbalancing the NADH excess in the cytosol. Probably linked to the different entry point of glycerol into metabolism, the supply of NADPH differed for this carbon source with respect to the reactions involved. Here, the oxidative PPP played only a minor role, whereas the mannitol cycle and the malic enzyme were recruited. For oleic acid the flux distribution differed drastically (Figure 7C). For optimal production, degradation involved two parallel routes, that in the mitochondrion as well as that in the glyoxysome resulting in a large relative flux through the glyoxylate shunt and reactions of the TCA cycle with the corresponding mitochondrial shuttle systems (Figure 7C). Additionally, the high supply of NADH by the degradation of the reduced fatty acids was obviously utilized by the mannitol cycle to form NADPH. The oxidative PPP was not involved in NADPH supply. Production on xylose demanded for increased NADPH supply, as indicated by average flux through the oxidative PPP (48 mol/mol hexose unit), the mannitol cycle (60 mol/mol hexose unit) and the malic enzyme (60 mol/mol hexose unit) (Figure 7D). This at least partly attributed to the NADPH demand linked to the xylose uptake system [38]. As for glucose, by-product formation was not observed for the alternative carbon sources under maximal production. The degree of reduction also played a role for the nitrogen source. The optimum yield decreased by about 18% for all carbon sources when nitrate was used instead of ammonia.

#### Prediction of amplification and deletion targets

**Figure 8:**
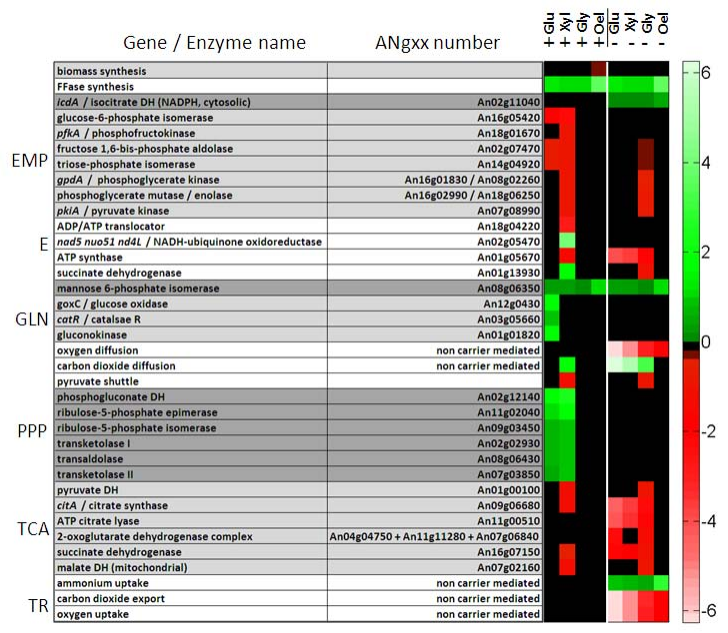
**Prediction of genetic targets for improved fructofuranosidase production in *A. niger *based on the target validity coefficient**. The target validity coefficient was obtained from correlation of flux through metabolic reactions with fructofuranosidase production flux within the calculated elementary modes. A positive value (green colour) relates to a reaction, which positively correlates with the production, whereas negative correlation is indicated by a negative value (red colour). Black colour indicates statistically insignificant values (no correlation = nc). The investigated biological scenarios comprise growth- (+) and non-growth-associated production (-) on glucose (Glu), glycerol (Gly), xylose (Xyl) and oleate (Ole) as carbon source. The absolute values for the target validity coefficients together with statistical information are additionally available in the supplementary material (Table A2 -- A7).

The reactions in the elementary modes were now screened for statistically significant correlation to the enzyme production. The potential of a metabolic reaction as genetic target was then expressed quantitatively whereby positive values denote amplification and negative values deletion targets, respectively. First investigations, considering the whole set of all 21,000 elementary modes, revealed only a few targets. A closer inspection revealed that most targets are specifically attributed to the cellular state. To exploit this observation systematically, the elementary modes were grouped into sub sets of growth-associated (simultaneous production of target protein and biomass) and non-growth-associated ones (production of target protein, no production of biomass) prior to analysis. Hereby, only modes with zero by-product formation were considered. This increased the hit rate of the approach substantially. The results for production on glucose, xylose, glycerol and oleic acid under growth-associated (+) and non-growth-associated conditions (-) are visualized as heat map (Figure 8). Fructofuranosidase synthesis and secretion and mannose 6-phosphate isomerase were identified as amplification targets independent of the biological state and also of the carbon source. These targets were also identified when all elementary modes were screened (data not shown). Other predicted targets strongly depended on the metabolic growth state. As example the amplification of the PPP and deletion/attenuation of the glycolysis display promising targets only under growth associated conditions. Cytosolic NADPH dependent isocitrate dehydrogenase, however, displayed a non-growth associated amplification target independent on the applied carbon source. Deletion or attenuation targets for non-growth conditions were found within the TCA cycle and also reactions linked to respiration and ATP metabolism. In comparison, no statistically valid correlations could be obtained for oleic acid as substrate in addition to the general findings. At this stage it appears that the underlying network for utilization of this complex substrate mixture is highly flexible and capable to achieve efficient production with significantly different underlying pathway usage.

Other target enzymes studied, including glucoamylase or epoxide hydrolase which differ in amino acid composition and glycosylation degree yielded rather similar targets for all metabolic scenarios studied.

## Discussion

Elementary mode analysis provides a rigorous basis to systematically characterize cellular phenotypes, metabolic flexibility and robustness which facilitates the understanding of cell physiology [39,40]. In the present work, this pathway analysis tool was applied and extended to predict systems-wide amplification and deletion targets in metabolic engineering towards improved bio-production in systems with optimally designed fluxes (FluxDesign). First evidence that the reactions derived here open realistic chances for improvement can be obtained from recent studies. An excellent test case is the very well studied *C. glutamicum*. From the targets predicted, various reactions have been successfully implemented towards superior production of lysine. This includes amplification of glucose 6-phosphate dehydrogenase [8], 6-phosphogluconate dehydrogenase [41], reactions within the lysine pathway [42] as well as product secretion [43], all shown to enhance lysine production Additionally, deletion of glucose 6-phosphate isomerase [44] or pyruvate dehydrogenase [45], have been successfully implemented into *C. glutamicum *for improved performance. Moreover, not yet validated targets such as the amplification of ammonium metabolism or reactions of the non-oxidative PPP or deletion/attenuation of TCA cycle reactions are predicted. For enzyme production in *A. niger*, much less metabolic engineering progress of central carbon metabolism is reported. The few studies available, however, illustrate that targets predicted here have proven valuable. As example, the amplification of the synthesis of glycosylation residues increased protein over-production [46,47]. Similarly, the amplification of the protein assembly route itself, has been shown to result in enhancement of production in *A. niger *[48]. Beyond, these experimental studies on more obvious targets, flux balance analysis and also stoichiometric flux analysis indicate the importance of sufficient NADPH supply for protein production in *A. niger *[49,21] and *A. oryzae *[21,50] whereby the PPP plays an important role which was also found in the present study.

The present approach did not reveal all relevant targets previously reported to redirect carbon flux. As example, the amplification of fructose bisphosphatase [9] or the deletion of phosphoenolpyruvate carboxykinase [51] both identified from ^13^C flux analysis as major targets for improved lysine production in *C. glutamicum*, was not predicted here. Still, the presented approach can be generally used to identify priority sorted amplification and deletion targets for metabolic engineering purposes under various conditions and thus displays a useful strategy to be combined with existing in silico tools [1] for strain engineering.

Due to the fact that elementary flux mode analysis enables the investigation of all possible physiological states in the cell, detailed insights into the underlying metabolism could be obtained. This includes the visualization of different flux states for optimum production which result from complementary pathways for the supply of NADPH (*A. niger*) or the regeneration of ATP (*C. glutamicum*). A closer inspection showed that this characteristic mainly originates from a small sub set of reactions, adding flexibility and robustness to the networks. The possibility to recruit different pathway modes for high production appears advantageous when approaching metabolic engineering strategies. Since it can be expected that certain genetic engineering strategies might not work for reasons of growth deficiency or undesired regulatory behaviour, the possibility to choose among different promising directions seems useful. Interestingly, the prediction of genetic targets depended on the metabolic state of the cell (Figure 7). Thus it turned out as relevant to focus the target search to a specific relevant scenario. Growing cells and non-growing cells pose different burdens on the metabolism, competing with product formation, so that different conclusions are derived. From practical perspective, both scenarios seem relevant, since for production were non-growing as well as growing cells can be applied [52,53]. The metabolic state is therefore an important point to be considered.

The models used in the present work are a condensed representation of the genome-wide metabolism relevant for the present study. Guided by the focus of the study we have considered industrially relevant substrates and clear objective products, whereas unusual substrates or other possible products appeared irrelevant here. It seems, however, easily possible to extend our approach to larger networks if desired, with additional substrates or even mixtures or also more detailed resolution of anabolic routes at the network periphery which were lumped here. The latter would, however, require a more detailed experimental basis on cellular composition as currently available.

## Conclusions

Combining elementary flux mode analysis with correlation of fluxes to desired network properties, potential amplification and deletion targets could be identified in industrially relevant production strains. Hereby, different scenarios considering the bioprocess environment or the metabolic state of the cell provided a detailed insight into the underlying pathway network. These findings appear very useful to guide strain engineers towards improved bio-production. This also might include a comparison among different potentially interesting hosts [12]. Admittedly, not every target predicted by FluxDesign will necessary lead to improved production, since stoichiometric modelling as applied here cannot consider e.g. cellular regulation or enzyme properties limiting or even blocking the desired network response towards targeted genetic perturbation. Still, the presented approach can be easily used to identify priority sorted amplification and deletion targets for metabolic engineering purposes under various conditions and thus displays a useful strategy to be incorporated into strain and bioprocess optimization.

## Abbreviations

6PG: 6-phosphogluconate; AC: acetate; AcCoA: acetyl-CoA; ADP: adenosine diphosphate; AKG: α-ketoglutarate; Asn: asparagine; ATP: adenosine triphosphate; CIT: citrate; CoA: coenzyme-A; CO2: carbon dioxide; DAP: dihydroxyacetone phosphate; CDW: cell dry weight; E4P: erythrose 4-phosphate; EC: enzyme commission; EFM: elementary flux mode; F6P: fructose 6-phosphate; FAD: flavin adenine dinucleotide (oxidized); FADH2: flavin adenine dinucleotide (reduced); FBP: fructose 1,6-bisphosphate; FFase: fructofuranosidase; FMM: fructose mannose metabolism; FOR: formate; FRC: fructose; FUM: fumarate; G6P: glucose 6-phosphate; Galf: galactofuranose; GAP: glyceraldehyde 3-phosphate; GLC: glucose; GlcNAc: N-acetyl-glucosamine; GLN: gluconate; GLP: glycerol 3-phosphate; GLY: glycerol; GLYN: glycerone; GOX: glyoxylate; H2S: hydrogen sulphide; ICT: isocitrate; LCoA: lenoate-CoA; LEN: lenoate; M6P: mannose 6-phosphate; MAL: malate; MAN: mannitol; MAP: mannitol 1-phosphate; MFA: metabolic flux analysis; NAD: nicotinamide adenine dinucleotide (oxidized); NADH: nicotinamide adenine dinucleotide (reduced); NADP: nicotinamide adenine dinucleotide phosphate (oxidized); NADPH: nicotinamide adenine dinucleotide phosphate (reduced); NH3: ammonia; NO3: nitrate; O2: oxygen; OAA: oxaloacetate; OCoA: oleate-CoA; OEL: oleate; OXA: oxalate; PEP: phosphoenolpyruvate; PPP: pentose phosphate pathway; PYR: pyruvate; R5P: ribose 5-phosphate; RUP: ribulose 5-phosphate; S7P: sedoheptulose 7-phosphate; SO4: sulphate; SUC: succinate; TCA: tricarboxylic acid; Q: ubiquinone ox.; QH2: ubiquinone red.; XOL: xylitol; XUP: xylulose 5-phosphate; XYL: xylose.

## Authors' contributions

GM created the metabolic models, designed the simulation experiments, performed the simulation studies, analysed the results, drafted all figures and assisted in drafting of the manuscript. ME programmed the post-processing toolbox for data processing. EFL contributed by discussions on the manuscript. CW supervised the work, designed the simulation experiments and drafted the paper. All authors read and approved the final manuscript.

## Supplementary Material

Additional file 1 **Scenario *Escherichia coli***. Small example network model of *E. coli *for succinyl-CoA production, results of the target validity calculation and statistical evaluation. Click here for file

Additional file 2 **Scenario *Corynebacterium glutamicum***. Metabolic network model of *C. glutamicum*, results of the target validity calculation and statistical evaluation. Click here for file

Additional file 3 **Scenario *Aspergillus niger***. Metabolic network model of *A. niger*, results of the target validity calculation and statistical evaluation. Click here for file

## References

[B1] Kim HU Kim TY Lee SY Metabolic flux analysis and metabolic engineering of microorganisms Mol Biosyst 2008 4 2 113 120 10.1039/b712395g 18213404

[B2] Suthers PF Burgard AP Dasika MS Nowroozi F Van Dien S Keasling JD Maranas CD Metabolic flux elucidation for large-scale models using ^13^C labeled isotopes Metab Eng 2007 9 5-6 387 405 10.1016/j.ymben.2007.05.005 17632026 PMC2121621

[B3] Patil KR Rocha I Forster J Nielsen J Evolutionary programming as a platform for in silico metabolic engineering BMC Bioinformatics 2005 6 308 10.1186/1471-2105-6-308 16375763 PMC1327682

[B4] Segre D Vitkup D Church GM Analysis of optimality in natural and perturbed metabolic networks Proc Natl Acad Sci USA 2002 99 23 15112 15117 10.1073/pnas.232349399 12415116 PMC137552

[B5] Trinh CT Unrean P Srienc F Minimal *Escherichia coli* cell for the most efficient production of ethanol from hexoses and pentoses Appl Environ Microbiol 2008 74 12 3634 3643 10.1128/AEM.02708-07 18424547 PMC2446564

[B6] Wittmann C Fluxome analysis using GC-MS Microb Cell Fact 2007 6 6 10.1186/1475-2859-6-6 17286851 PMC1805451

[B7] Pharkya P Maranas CD An optimization framework for identifying reaction activation/inhibition or elimination candidates for overproduction in microbial systems Metab Eng 2006 8 1 1 13 10.1016/j.ymben.2005.08.003 16199194

[B8] Becker J Klopprogge C Herold A Zelder O Bolten CJ Wittmann C Metabolic flux engineering of L-lysine production in *Corynebacterium glutamicum*--over expression and modification of G6P dehydrogenase J Biotechnol 2007 132 2 99 109 10.1016/j.jbiotec.2007.05.026 17624457

[B9] Becker J Klopprogge C Zelder O Heinzle E Wittmann C Amplified expression of fructose 1,6-bisphosphatase in Corynebacterium glutamicum increases in vivo flux through the pentose phosphate pathway and lysine production on different carbon sources Appl Environ Microbiol 2005 71 12 8587 8596 10.1128/AEM.71.12.8587-8596.2005 16332851 PMC1317465

[B10] Wang L Birol I Hatzimanikatis V Metabolic control analysis under uncertainty: framework development and case studies Biophys J 2004 87 6 3750 3763 10.1529/biophysj.104.048090 15465856 PMC1304888

[B11] Trinh CT Wlaschin A Srienc F Elementary mode analysis: a useful metabolic pathway analysis tool for characterizing cellular metabolism Appl Microbiol Biotechnol 2009 81 5 813 826 10.1007/s00253-008-1770-1 19015845 PMC2909134

[B12] Krömer JO Wittmann C Schröder H Heinzle E Metabolic pathway analysis for rational design of L-methionine production by *Escherichia coli* and *Corynebacterium glutamicum* Metab Eng 2006 8 4 353 369 10.1016/j.ymben.2006.02.001 16621639

[B13] Carlson R Fell D Srienc F Metabolic pathway analysis of a recombinant yeast for rational strain development Biotechnol Bioeng 2002 79 2 121 134 10.1002/bit.10305 12115428

[B14] Notebaart RA B T Siezen RJ Papp B Co-Regulation of Metabolic Genes Is Better Explained by Flux Coupling Than by Network Distance PLoS Comput Biol 2008 4 e26 10.1371/journal.pcbi.0040026 18225949 PMC2211535

[B15] Leuchtenberger W Huthmacher K Drauz K Biotechnological production of amino acids and derivatives: current status and prospects Appl Microbiol Biotechnol 2005 69 1 1 8 10.1007/s00253-005-0155-y 16195792

[B16] Kjeldsen KR Nielsen J In silico genome-scale reconstruction and validation of the *Corynebacterium glutamicum* metabolic network Biotechnol Bioeng 2008 102 583 597 10.1002/bit.22067 18985611

[B17] Wittmann C Becker J The L-lysine story: From metabolic pathways to industrial production Microbiology Monographs 2007 Springer Berlin/Heidelberg

[B18] Jones MG The first filamentous fungal genome sequences: *Aspergillus* leads the way for essential everyday resources or dusty museum specimens? Microbiology 2007 153 Pt 1 1 6 10.1099/mic.0.2006/001479-0 17185529

[B19] Andersen MR Nielsen ML Nielsen J Metabolic model integration of the bibliome, genome, metabolome and reactome of *Aspergillus niger* Mol Syst Biol 2008 4 178 10.1038/msb.2008.12 18364712 PMC2290933

[B20] Zuccaro A Gotze S Kneip S Dersch P Seibel J Tailor-made fructooligosaccharides by a combination of substrate and genetic engineering Chembiochem 2008 9 1 143 149 10.1002/cbic.200700486 18058889

[B21] Pedersen H Christensen B Hjort C Nielsen J Construction and characterization of an oxalic acid nonproducing strain of *Aspergillus niger* Metab Eng 2000 2 1 34 41 10.1006/mben.1999.0136 10935933

[B22] Naundorf A Melzer G Archelas A Furstoss R Wohlgemuth R Influence of pH on the expression of a recombinant epoxide hydrolase in *Aspergillus niger* Biotechnol J 2009 4 5 756 765 10.1002/biot.200900034 19452475

[B23] Schuster S Hilgetag C On elementary flux modes in biochemical reaction systems at steady state Journal of Biological Systems 1994 2 165 182 10.1142/S0218339094000131

[B24] Schuster S Dandekar T Fell DA Detection of elementary flux modes in biochemical networks: a promising tool for pathway analysis and metabolic engineering Trends Biotechnol 1999 17 2 53 60 10.1016/S0167-7799(98)01290-6 10087604

[B25] Wagner C Nullspace approach to determine the elementary modes of chemical reaction systems Journal of Physical Chemistry B 2004 108 7 2425 2431 10.1021/jp034523f

[B26] Terzer M Stelling J Large-scale computation of elementary flux modes with bit pattern trees Bioinformatics 2008 24 19 2229 2235 10.1093/bioinformatics/btn401 18676417

[B27] Bernstein JA Khodursky AB Lin PH Lin-Chao S Cohen SN Global analysis of mRNA decay and abundance in *Escherichia coli *at single-gene resolution using two-color fluorescent DNA microarrays Proc Natl Acad Sci USA 2002 99 15 9697 9702 10.1073/pnas.112318199 12119387 PMC124983

[B28] Butland G Babu M Diaz-Mejia JJ Bohdana F Phanse S Gold B Yang W Li J Gagarinova AG Pogoutse O eSGA: *E. coli* synthetic genetic array analysis Nat Methods 2008 5 9 789 795 10.1038/nmeth.1239 18677321

[B29] Wittmann C de Graaf A Eggeling L, Bott M Metabolic flux analysis in *Corynebacterium glutamicum* Handbook of Corynebacterium glutamicum 2005 Boca Raton: CRC Press 277 304

[B30] David H Åkesson M Nielsen J Reconstruction of the central carbon metabolism of *Aspergillus niger* European Journal of Biochemistry 2003 270 21 4243 4253 10.1046/j.1432-1033.2003.03798.x 14622289

[B31] Sun J Zeng AP IdentiCS--identification of coding sequence and *in silico *reconstruction of the metabolic network directly from unannotated low-coverage bacterial genome sequence BMC Bioinformatics 2004 5 112 10.1186/1471-2105-5-112 15312235 PMC514700

[B32] Deshpande N Wilkins MR Packer N Nevalainen H Protein glycosylation pathways in filamentous fungi Glycobiology 2008 18 8 626 637 10.1093/glycob/cwn044 18504293

[B33] Bier DM The energy costs of protein metabolism: lean and mean on Uncle Sam's team The Role of Protein and Amino Acids in Sustaining and Enhancing Performance 1999 Washington, DC: National Academy Press 109 119

[B34] Apweiler R Hermjakob H Sharon N On the frequency of protein glycosylation, as deduced from analysis of the SWISS-PROT database Biochim Biophys Acta 1999 1473 1 4 8 10580125 10.1016/s0304-4165(99)00165-8

[B35] Trimble RB Atkinson PH Structure of yeast external invertase Man8-14GlcNAc processing intermediates by 500-megahertz 1H NMR spectroscopy J Biol Chem 1986 261 21 9815 9824 3525534

[B36] Pel HJ de Winde JH Archer DB Dyer PS Hofmann G Schaap PJ Turner G de Vries RP Albang R Albermann K Genome sequencing and analysis of the versatile cell factory *Aspergillus niger *CBS 513.88 Nat Biotechnol 2007 25 2 221 231 10.1038/nbt1282 17259976

[B37] Williamson G Belshaw JP Williamson MP O-glycosylation in *Aspergillus* glucoamylase. Conformation and role in binding Biochem 1992 282 423 428 10.1042/bj2820423 PMC1130795 1546955

[B38] Prathumpai W Gabelgaard JB Wanchanthuek P Vondervoort PJ van de de Groot MJ McIntyre M Nielsen J Metabolic control analysis of xylose catabolism in *Aspergillus* Biotechnol Prog 2003 19 4 1136 1141 10.1021/bp034020r 12892473

[B39] Papin JA Stelling J Price ND Klamt S Schuster S Palsson BO Comparison of network-based pathway analysis methods Trends Biotechnol 2004 22 8 400 405 10.1016/j.tibtech.2004.06.010 15283984

[B40] Schuster S Hilgetag C Woods JH Fell DA Reaction routes in biochemical reaction systems: algebraic properties, validated calculation procedure and example from nucleotide metabolism J Math Biol 2002 45 2 153 181 10.1007/s002850200143 12181603

[B41] Ohnishi J Katahira R Mitsuhashi S Kakita S Ikeda M A novel *gnd *mutation leading to increased L-lysine production in *Corynebacterium glutamicum* FEMS Microbiol Lett 2005 242 2 265 274 10.1016/j.femsle.2004.11.014 15621447

[B42] Eggeling L Oberle S Sahm H Improved L-lysine yield with *Corynebacterium glutamicum*: use of *dapA *resulting in increased flux combined with growth limitation Appl Microbiol Biotechnol 1998 49 1 24 30 10.1007/s002530051132 9487706

[B43] Broer S Eggeling L Kramer R Strains of *Corynebacterium glutamicum *with Different Lysine Productivities May Have Different Lysine Excretion Systems Appl Environ Microbiol 1993 59 1 316 321 16348855 10.1128/aem.59.1.316-321.1993 PMC202097

[B44] Marx A Hans S Mockel B Bathe B de Graaf AA Metabolic phenotype of phosphoglucose isomerase mutants of *Corynebacterium glutamicum* J Biotechnol 2003 104 1-3 185 197 10.1016/S0168-1656(03)00153-6 12948638

[B45] Blombach B Schreiner ME Moch M Oldiges M Eikmanns BJ Effect of pyruvate dehydrogenase complex deficiency on L-lysine production with *Corynebacterium glutamicum* Appl Microbiol Biotechnol 2007 76 3 615 623 10.1007/s00253-007-0904-1 17333167

[B46] Jacobs DI Olsthoorn MM Maillet I Akeroyd M Breestraat S Donkers S Hoeven RA van der Hondel CA van den Kooistra R Lapointe T Effective lead selection for improved protein production in *Aspergillus niger* based on integrated genomics Fungal Genet Biol 2008 46 Suppl 1 (1) S141 152 18824119 10.1016/j.fgb.2008.08.012

[B47] Brink HJ van den Petersen SG Rahbek-Nielsen H Hellmuth K Harboe M Increased production of chymosin by glycosylation J Biotechnol 2006 125 2 304 310 10.1016/j.jbiotec.2006.02.024 16621086

[B48] Moralejo FJ Cardoza RE Gutierrez S Martin JF Thaumatin production in *Aspergillus awamori* by use of expression cassettes with strong fungal promoters and high gene dosage Appl Environ Microbiol 1999 65 3 1168 1174 10049878 10.1128/aem.65.3.1168-1174.1999 PMC91159

[B49] Melzer G Dalpiaz A Grote A Kucklick M Göcke Y Jonas R Dersch P Franco-Lara E Nörtemann B Hempel DC Metabolic flux analysis using stoichiometric models for *Aspergillus niger*: comparison under glucoamylase-producing and non-producing conditions J Biotechnol 2007 132 4 405 417 10.1016/j.jbiotec.2007.08.034 17931730

[B50] Schmidt K Norregaard LC Pedersen B Meissner A Duus JO Nielsen JO Villadsen J Quantification of intracellular metabolic fluxes from fractional enrichment and 13C-13C coupling constraints on the isotopomer distribution in labeled biomass components Metab Eng 1999 1 2 166 179 10.1006/mben.1999.0114 10935929

[B51] Riedel C Rittmann D Dangel P Mockel B Petersen S Sahm H Eikmanns BJ Characterization of the phosphoenolpyruvate carboxykinase gene from *Corynebacterium glutamicum *and significance of the enzyme for growth and amino acid production J Mol Microbiol Biotechnol 2001 3 4 573 583 11565516

[B52] Mills DA Flickinger MC Cloning and sequence analysis of the meso-diaminopimelate decarboxylase gene from *Bacillus methanolicus *MGA3 and comparison to other decarboxylase genes Appl Environ Microbiol 1993 59 9 2927 2937 8215365 10.1128/aem.59.9.2927-2937.1993 PMC182388

[B53] Flickinger MC Rouse MP Sustaining protein synthesis in the absence of rapid cell division: an investigation of plasmid-encoded protein expression in *Escherichia coli* during very slow growth Biotechnol Prog 1993 9 6 555 572 10.1021/bp00024a001 7764344

