# Supplementary material for: Flux Design: In silico design of cell factories based on correlation of pathway fluxes to desired properties
Source: BMC Syst Biol. 2009 Dec 25;3:120. doi: 10.1186/1752-0509-3-120 (PMC2808316; doi:10.1186/1752-0509-3-120)
Supplement: Additional file 1 — Scenario Escherichia coli. Small example network model of E. coli for succinyl-CoA production, results of the target validity calculation and statistical evaluation. [file 1752-0509-3-120-S1.DOC]

**Supplemental material 1 – Example network**

The reaction scheme in Figure C1 includes the tricarboxylic acid (TCA) cycle, glyoxylate shunt and adjacent amino acid metabolism. In this example, all cofactors, such as ATP and NAD+ were considered as external, as are 2-phospho-glycerate (PG), NH3 and CO2. A detailed description of the enzymes and metabolites is given in the legend of figure C1.

The metabolic network for the demonstration of the proposed method was derived from Schuster et al. [1].

Figure C1: Reaction scheme consisting of the tricarboxylic acid cycle, glyoxylate shunt and some adjacent reactions of amino acid metabolism in *Escherichia coli*. Abbreviations of metabolites: AcCoA, acetyl-CoA; Ala, alanine; Asp, aspartate; Cit, citrate; Fum, fumarate; Glu, glutamate; Gly, glyoxylate; IsoCit, isocitrate; Mal, malate; OAA, oxaloacetate; OG, 2-oxoglutarate; PEP, phosphoenolpyruvate; PG, 2-phosphoglycerate; Pyr, pyruvate; Succ, succinate; SucCoA, succinyl-CoA. Abbreviations of enzymes: AceEF, pyruvate dehydrogenase; Acn, aconitase; AspA, aspartase; AspC, aspartate aminotransferase; Eno, enolase; Fum, fumarase; Gdh, glutamate dehydrogenase; GltA, citrate synthase; Icd, isocitrate dehydrogenase (in *E. coli* with cofactors NADP/NADPH); Icl, isocitrate lyase; Mas, malate synthase; IlvE/AvtA, branched-chain amino acid aminotransferase/valine-pyruvate aminotransferase; Mdh, malate dehydrogenase; Pck, PEP carboxykinase (in *E. coli* with cofactors ADP/ATP); Ppc, PEP carboxylase; Pps, PEP synthetase; Pyk, pyruvate kinase; Sdh, succinate dehydrogenase; SucAB, 2-oxoglutarate dehydrogenase; SucCD, succinyl-CoA synthetase (in *E. coli* with cofactors ADP/ATP); AlaCon, AspCon, GluCon and SucCoACon, consumption of alanine, aspartate, glutamate and succinyl-CoA, respectively. Reversible reactions are indicated by double arrow-heads.

All 16 elementary modes (for detail see [1]) were transformed in matrix notation:

The reactions (enzymes) are presented in the columns, the elementary modes in the rows.

For the calculation of target validity for succinyl-CoA production (SucCoACon), only elementary flux modes with succinyl-CoA production were considered, which means that all rows with a zero entry in column SucCoACon were deleted. Hence, the matrix dimension decreased from a dimension 16 x 24 matrix (16 elementary modes; 24 reactions) to a 6 x 24 matrix, whereas only the elementary modes {8, 9, 10, 11, 13, 16} were further used for the calculation.

The coefficients **s**_i,j_ of reactions **i** of each elementary mode **j** are normalized to the substrate coefficients **s**_C,j_ (Eno) leading to the yield coefficients **ν_i,j_.** The modes have been arranged with increasing size of the succinyl-CoA yields **ν_SucCoACon_** (reaction: SucCoACon) as shown in following matrix.

The formation of interesting substances from succinyl-CoA can occur via the six different modes {8, 9, 10, 11, 13, 16}, whereas no co-production of glutamate, alanine and aspartate were observed. The modes {8, 10} offered the highest maximal theoretical yields for succinyl-CoA.

Each of **ν_i_** were correlated as function of **ν_SucCoACon_** resulting in statistical evident correlations or not (Table C2).

**Statistical evaluation**

**Table C2:** Statistical analysis of simulation data for succinyl-CoA production with *E. coli*. R²: regression coefficient, alpha: slope-correlation coefficient, NOSTAT: no statistical evaluation. The values correspond to Figure 2a. The entries of ‘#DIV/0’ regarded to constant values (or complete zeros) of stoichiometric coefficients for the corresponding enzyme.

**Reference**

1. Schuster S, Dandekar T, Fell DA**: Detection of elementary flux modes in biochemical networks: a promising tool for pathway analysis and metabolic engineeri**ng*. Trends Biotechno*l 1999**,** 17(2):53-60.
