# Supplementary material for: Flux Design: In silico design of cell factories based on correlation of pathway fluxes to desired properties
Source: BMC Syst Biol. 2009 Dec 25;3:120. doi: 10.1186/1752-0509-3-120 (PMC2808316; doi:10.1186/1752-0509-3-120)
Supplement: Additional file 2 — Scenario Corynebacterium glutamicum. Metabolic network model of C. glutamicum, results of the target validity calculation and statistical evaluation. [file 1752-0509-3-120-S2.DOC]

**Supplemental material 2 – *Corynebacterium glutamicum***

The metabolic reactions of the metabolic model of *Corynebacterium glutamicum* for lysine production were derived from the recently published genome scale metabolic model [1]. The biomass composition was taken from Marx et al. [2].

**Table B1: Stoichiometric equations of the metabolic model of *C. glutamicum*.** The units of stoichiometric coefficients are in “mol” if not other mentioned in brackets beside the subheadings. ‘-->’ indicates irreversible reaction, ‘<==>’ indicates reversible reaction.

| **Description** | **Metabolic reactions** |
| --- | --- |
|  | **Transport reactions** |
| glucose import | --> GLC[e] |
| biomass export | biomass[c] --> |
| lysine export | LYS[c] --> |
| maintenance export | ATPmaintenance[c] --> |
| sulfate import | --> SO4[e] |
| carbon dioxide export | CO2[c] --> |
| oxygen import | --> O2[c] |
| ammonium import | --> NH3[c] |
| maintenance | ATP[c] --> ADP[c] + ATPmaintenance[c] |
|  |  |
|  |  |
| **gene name / ORF** | **Embden-Meyerhoff-Parnas pathways / Gluconeogenesis** |
| GLC_in_PEP / Q45298 | PEP[c] + GLC[e] --> PYR[c] + G6P[c] |
| pgi / Q8NS31 | G6P[c] <==> F6P[c] |
| pfkA / Q8NR14 | ATP[c] + F6P[c] --> ADP[c] + F-16-BP[c] |
| fda / P19537 | F-16-BP[c] <==> GA3P[c] + DAHP[c] |
| tpiA / P19583 | DHAP[c] <==> GA3P[c] |
| gapA / Q01651 | GA3P[c] + NAD[c] <==> 13-PG[c] + NADH[c] |
| pgk / Q01655 | ADP[c] + 13-PG[c] --> ATP[c] + 3-PG[c] |
| pgm / Q8NTA5 | 3-PG[c] <==> 2-PG[c] |
| eno / Q8NRS1 | 2-PG[c] <==> PEP[c] |
| pyk / Q46078 | PEP[c] + ADP[c] --> PYR[c] + ATP[c] |
|  | F-16-BP[c] --> F6P[c] |
|  |  |
|  | **Pentose-Phosphate Pathway** |
| zwf / Q6M517 | G6P[c] + NADP[c] --> GLC-LAC[c] + NADPH[c] |
| opcA | GLC-LAC[c] --> 6-P-Gluconate[c] |
| gnd / Q8NQI2 | 6-P-Gluconate[c] + NADP[c] --> RIB-5P[c] + CO2[c] + NADPH[c] |
| rpe / Q8NQ49 | RIB-5P[c] <==> XYL-5P[c] |
| rpi / Q8NMZ0 | RIB-5P[c] <==> RIBO-5P[c] |
| tkt_1 / Q6M519 | S7P[c] + GA3P[c] <==> RIBO-5P[c] + XYL-5P[c] |
| tal / Q8NQ64 | S7P[c] + GA3P[c] <==> E-4P[c] + F6P[c] |
| tkt_2 / Q8NQ65 | F6P[c] + GA3P[c] <==> E-4P[c] + XYL-5P[c] |
|  |  |
|  | **Citrate Cycle** |
| aceE / Q8NNF6 | PYR[c] + H-CoA[c] + NAD[c] --> AC-CoA[c] + NADH[c] + CO2[c] |
| gltA / P42457 | AC-CoA[c] + OA[c] --> CIT[c] + H-CoA[c] |
| acn / Q8NQ98 | CIT[c] <==> Cis-ACO[c] |
| acn / Q8NQ98 | Cis-ACO[c] <==> ICI[c] |
| icd / P50216 | ICI[c] + NADP[c] --> AKG[c] + CO2[c] + NADPH[c] |
| odhA/ sucB / lpd / P96746 / Q8NNJ2 / Q8NSI4 | AKG[c] + NAD[c] + H-CoA[c] --> SUCC-CoA[c] + NADH[c] + CO2[c] |
| sucD / Q8NMK8 | SUCC-CoA[c] + ADP[c] --> SUCC[c] + H-CoA[c] + ATP[c] |
| sdhCAB / Q8NMK7 | SUCC[c] + MK[c] <==> FUM[c] + MKH2[c] |
| fumC / Q8NRN8 | FUM[c] <==> MAL[c] |
| mdh / Q8NN33 | MAL[c] + NAD[c] --> OA[c] + NADH[c] |
|  |  |
|  | **Glyoxylate Cycle** |
| aceA / P42449 | ICI[c] --> GLYOXY[c] + SUCC[c] |
| aceB | GLYOXY[c] + AC-CoA[c] --> MAL[c] + H-CoA[c] |
|  |  |
|  | **Anaplerotic reactions** |
| pyc / O54587 | PYR[c] + ATP[c] + CO2[c] --> OA[c] + ADP[c] |
| ppc / P12880 | PEP[c] + CO2[c] --> OA[c] |
| pckG / Q9AEM1 | OA[c] + ATP[c] --> PEP[c] + ADP[c] + CO2[c] |
| mez / Q8NLD5 | MAL[c] + NADP[c] --> PYR[c] + CO2[c] + NADPH[c] |
|  |  |
|  | **Amino acid metabolism** |
| gdh / P31026 | AKG[c] + NH3[c] + NADPH[c] --> GLU[c] + NADP[c] |
| aspB / Q6M8B5 | OA[c] + GLU[c] <==> ASP[c] + AKG[c] |
| lysC / P26512 | ASP[c] + ATP[c] --> ASP-P[c] + ADP[c] |
| asd / P26511 | ASP-P[c] + NADPH[c] --> ASP-SA[c] + NADP[c] |
| dapA / P19808 | ASP-SA[c] + PYR[c] --> DHP[c] |
| dapB / P40110 | DHP[c] + NADPH[c] --> THDP[c] + NADP[c] |
| dapD | THDP[c] + SUCC-CoA[c] --> SAP[c] + H-CoA[c] |
| dapC/ Q6M8B5 | SAP[c] + GLU[c] --> SADP[c] + AKG[c] |
| dapE / Q59284 | SADP[c] --> SUCC[c] + DAP[c] |
| ddh / P04964 | THDP[c] + NADPH[c] + NH3[c] --> DAP[c] + NADP[c] |
| lysA / P09890 | DAP[c] --> LYS[c] + CO2[c] |
|  |  |
|  | **Energy metabolism** |
|  | NADH[c] + (0.5) O2[c] + (2) ADP[c] --> NAD[c] + (2) ATP[c] |
|  | MKH2[c] + (0.5) O2[c] + (2) ADP[c] --> MK[c] + (2) ATP[c] |
| adk_1 / P49973 | AMP[c] + ATP[c] --> (2) ADP[c] |
|  |  |
|  | **Biomass synthesis (mmol/g dry weight)** |
| biomass synthesis | (6.231) NH3[c] + (0.233) H2S[c] + (0.205) G6P[c] + (0.071) F6P[c] + (0.879) RIBO-5P[c] + (0.268) E-4P[c] + (0.129) GA3P[c] + (1.295) 3-PG[c] + (0.534) PEP[c] + (1.807) PYR[c] + (2.5) AC-CoA[c] + (1.71) OA[c] + (1.252) AKG[c] + (14.849) NADPH[c] + (29.2) ATP[c] + (3.111) NAD[c] --> biomass[c] + (14.849) NADP[c] + (2.5) H-CoA[c] + (2.537) CO2[c] + (29.2) ADP[c] + (3.111) NADH[c] |
|  |  |
|  | **Sulphate metabolism** |
| cysND / Q8NLX1 | SO4[e] + ATP[c] --> SO4[c] + ADP[c] |
| cysH_1 Q6M242 | SO4[c] + (2) ATP[c] + NADPH[c] --> H2SO3[c] + ADP[c] + AMP[c] + NADP[c] |
| cysI | H2SO3[c] + (3) NADPH[c] --> H2S[c] + (3) NADP[c] |

**Abbreviations**

**Table B2:** Abbreviations of metabolites.

| GLC | glucose |
| --- | --- |
| O2 | oxygen |
| CO2 | carbon dioxide |
| LYS | lysine |
| NH3 | ammonium |
| PEP | phosphoenolpyruvate |
| PYR | pyruvate |
| G6P | glucose 6-phosphate |
| GLC-LAC | gluconolactone |
| XYL-5P | xylose 5-phosphate |
| E-4P | erythrose 4-phosphate |
| F6P | fructose 6-phosphate |
| S7P | sedoheptulose 7-phosphate |
| GA3P | glyceraldehyde 3-phosphate |
| DHAP | dihydroxyacetone phosphate |
| F-16-BP | fructose 1,6-bis-phosphate |
| RIBO-5P | ribulose 5-phosphate |
| MAL | malate |
| FUM | fumarate |
| MK | menaquinol ox. |
| AKG | 2-oxo glutarate |
| AC-CoA | acetyl-CoA |
| CIT | citrate |
| SUCC-CoA | succinyl-CoA |
| H-CoA | coenzyme A |
| SUCC | succinate |
| ICI | isocitrate |
| Cis-ACO | cis-aconitate |
| MKH2 | menaquinol red. |
| GLYOXY | glyoxylate |
| OA | oxalacetate |
| GLU | glutamate |
| ASP | aspartate |
| ASP-P | aspartyl-phosphate |
| ASP-SA | aspartyl-semialdehyde |
| DHP | dehydropicolinate |
| SAP | succinyl-2,6-amino-6-ketopimelate |
| THDP | L(delta-1)-Piperideine-2,6-dicarboxylate |
| SADP | N-succinyl-L-2,6-diaminopimelate |
| DAP | L,L-2,6-Diaminopimelate |

**Statistical evaluation**

**Table B3:** Statistical analysis of simulation data for lysine production with *C. glutamicum*. R²: regression coefficient, alpha: slope-correlation coefficient, NOSTAT: no statistical evaluation. The values correspond to Figure 3. The entries of ‘#DIV/0’ regarded to constant values (or complete zeros) of stoichiometric coefficients for the corresponding enzyme.

**Reference**

1. Kjeldsen KR, Nielsen J: **In silico genome-scale reconstruction and validation of the Corynebacterium glutamicum metabolic network**. *Biotechnol Bioeng* 2009, **102**(2):583-597.

2. Marx A, de Graaf AA, Wiechert W, Eggeling L, Sahm H: **Determination of the fluxes in the central metabolism of *Corynebacterium glutamicum* by nuclear magnetic resonance spectroscopy combined with metabolite balancing**. *Biotechnol Bioeng* 1996, **49**(2):111-129.
