# Supplementary material for: Flux Design: In silico design of cell factories based on correlation of pathway fluxes to desired properties
Source: BMC Syst Biol. 2009 Dec 25;3:120. doi: 10.1186/1752-0509-3-120 (PMC2808316; doi:10.1186/1752-0509-3-120)
Supplement: Additional file 3 — Scenario Aspergillus niger. Metabolic network model of A. niger, results of the target validity calculation and statistical evaluation. [file 1752-0509-3-120-S3.DOC]

**Supplemental material 3 – *Aspergillus niger***

In the following, the metabolic reactions of the metabolism of *A. niger* were derived from the recently published genome scale metabolic model by Andersen et al. [1]. Also literature information of David et al. [2] Diano et al. [3] and Pel et al. [4] were considered if not other references were cited.

**Transport processes.** Monosaccharide (glucose, xylose) uptake by the cell (R40, R99) is carried out by a mono-saccharide transporter with high affinity [5].

Pyruvate is transported intracellularly between mitochondrial and cytosolic compartments via the pyruvate shuttle (R24). Additionally, the citrate/malate (R25), fumarate (R26), isocitrate/malate (R29) and succinate (R28) shuttle were considered [1, 2]. ATP can be transported via an ATP/ADP translocator (R23) localized in the mitochondrial membrane [1, 4]. Finally, succinate (R132), isocitrate (R130), malate (R134) and AcCoA (R135) are allowed to pass through the glyoxysomal membrane [2, 4, 6].

**Embden-Meyerhoff-Parnas (EMP) and pentose-phosphate pathway***.* The enzymes for EMP and for the pentose-phosphate pathway (PPP) take place exclusively in the cytosol of the cell. Glucose is phosphorylated by ATP in a reaction catalysed by hexokinase (R41) [7]. The further degradation of D-glucose 6-phosphate to pyruvate takes place through the well known Embden-Meyerhoff pathway (EMP), which include 7 reactions (R42, R43, R44, R45, R46, R47, R48).

The channelling of the D-xylose carbon scaffold into the glycolytic pathway occurs through the non-oxidative part of the pentose phosphate pathway (PPP). Xylose is converted intracellular into the polyol xylitol by the NADPH-dependent polyol dehydrogenase (R100), which is then converted into D-xylulose by the NADH-dependent xylitol dehydrogenase (R101) [8].

The intracellular pool of xylulose is assumed to be phosphorylated by the ATP-depending xylulokinase (R102) producing xylulose-5-phosphate. Two molecules of xylulose-5-phosphate enter the non-oxidative part of PPP and are converted together with 1 molecule of ribose 5-phosphate (originating from the oxidative part of the PPP) by interlinked reactions catalysed by a transketolase (R78, R80) and a transaldolase (R79) into 2 molecules of fructose 6-phosphate and 1 of glyceraldehydes 3-phosphate.

The oxidative part of the PPP (R74, R75) generates reducing equivalents, in the form of NADPH, for reductive biosynthesis reactions within cells. Furthermore, it provides precursor in the form of ribose-5-phosphate, for the synthesis of the nucleotides and nucleic acids with the simultaneously release of carbon dioxide.

**Fructose and mannose metabolism.** Fructose can be phosphorylated by a hexokinase into fructose 6-phosphate (R57). Mannose 6-phosphate can be formed by isomerisation of fructose 6-phosphate (R55) and further used for the glycosylation of proteins. Fructose can be converted into mannitol by the reversible NADPH-depending mannitol 2-dehydrogenase (R53) in the cytosolic part of the cell. Mannitol as well as xylitol (see PPP) plays important roles in the prevention of oxidative stress, osmotic regulation and the provision of storage carbon sources. The production of these polyols has been described in recent work [3].

**Anaplerotic reactions, citrate cycle, glyoxylate and dicarboxylate reactions.** The citrate cycle has been described in detail [9] and was incorporated into the stoichiometric model (R141, R142, R143, R144, R145, R146, R147, R148). The pyruvate carboxylase reaction is assumed to be localized not only in the cytosol (R66) [10] but also in the mitochondrion (R151) [11]. The carbon dioxide used in this anaplerotic reaction, yielding mitochondrial oxaloacetate, comes from the decarboxylation of pyruvate by the pyruvate dehydrogenase complex (R140), which is localized in the mitochondrion. On the other hand, the oxaloacetate formed by the cytosolic pyruvate carboxylase is converted to malate by malate dehydrogenase (R86) and thereupon transported into the mitochondria (R25) [12]. Therefore, malate serves an essential function of replenishing the metabolite pool of the citrate cycle and, in particular, oxaloacetate serves as precursor for biomass (R220) as well as for fructofuranosidase synthesis (R210).

The cytosolic oxaloacetate could be also transformed into phosphoenolpyruvate by the ATP-dependent phosphoenolpyruvate-carboxykinase (R65). Finally, it was assumed that the oxaloacetate-hydrolase (R87) is localized in the cytosol and is responsible for the production of oxalic acid, which accumulates in the culture medium [13]. Although, oxalic acid can be hydrolysed (R89) into formate and CO2 and subsequently oxidized by the formate dehydrogenase (R90) forming CO2 which is coupled with NADH generation. However, in the outmost cases, oxalic acid will be transported into the extra-cellular medium (R9).

As mentioned earlier, the generation of cytosolic NADPH deliver essential energy for the biosynthesis of numerous precursors for biomass and product synthesis, e.g. amino acids, nucleotides and fatty acids. Beside the oxidative part of the PPP, two further reactions have to be considered in the metabolic model, which are responsible for NADPH generation. The NADP-depending isocitrate dehydrogenase, which is localized not only in the mitochondrion (R144), but also in the cytosolic part (R93) forming α-ketoglutarate, a precursor for biomass and product synthesis, was considered in the model.

The malic enzyme (R85), also known as decarboxylating malate dehydrogenase, utilizes NADP to catalyze the oxidative decarboxylation of malate to pyruvate and carbon dioxide. Finally, the reactions of the glyoxysomal cycle are considered in the model (R131, R133).

**Energy metabolism.** The oxidative metabolism of substrates takes place in the mitochondria. For ATP production in the respiratory chain a P/O ratio of 2.64 for NADH (R149) and 1.64 for succinate (R150) and cytosolic NADH was considered [1].

The inter-conversion of NADPH into NADH occurs via the transhydrogenase reaction (R97) in the cytosol [4]. However, the formation of NADPH from NADH is linked with an ATP consumption, which occurs only in the cytosol. This assumption was made under considering that the mannitol-cycle plays an important role in NADPH production, which was proposed for the fungus *Alternaria alternata* [14] and for *Aspergillus* *sp.* [15].

The net result of mannitol cycle (R53, R54, R56) is a transhydrogenase activity with a net consumption of ATP (NADH + NADP + ATP --> NAD + NADPH + ADP).

Cause of the compartmentalization, the ATP must be transported across the mitochondrial membrane, which is realized by an ATP/ADP-translocation (R23).

The assimilation of sulphate comprises uptake (R5) and subsequent reduction into hydrogen-sulphide (R170), whereas the latter include two subsequent reactions: reduction of sulphate to sulphite requiring 2 mol ATP and 1 mol NADPH and the reduction of sulphite to sulphide requiring 3 mol NADPH.

**Lipid metabolism.** Extracellular lipases hydrolyse triglycerides to fatty acids and glycerol. They can produced extracellularly by *A. niger* when different complex triglycerides are supplied [16]. The degradation of fatty acids in *Aspergillus sp.* occurred both in the mitochondria but also in the peroxisomes / glyoxysomes. The metabolic reactions (R180 – R198) in the metabolic model were applied according to literature data [17].

**Format of reaction model.** Listed in what follows (Table A) is the metabolic reaction model in Palsson-like formula with compartments. Reactions with arrow ‘**-->**’ are irreversible and reactions with double arrow ‘<==>’ are reversible with respect to the thermodynamic constraints. External metabolites are indicated by the short-cut ‘[ext]’. The metabolic model included the compartmentalization with cytosol, extracellular area, golgi and mitochondrion indicated by [c], [e], [g] and [m], respectively. The stoichiometric coefficients are listed in parenthesis when the values are ≠ 1.

**Table A1: Stoichiometric equations of the metabolic model.** The reactions are either specific for a certain carbon source (“G”: glucose, “g”: glycerol, “S”: soybean oil, “X”: xylose), or relevant for all carbon sources (“for all”), which is indicated in the left column. The units of stoichiometric coefficients are in “mol” if not other mentioned in brackets beside the subheadings.

| Network | **Transport Systems** | | | | | |
| --- | --- | --- | --- | --- | --- | --- |
|  | | | | | |
| G | R1: | glucose [ext] --> glucose[e] | | |  | |
| for all | R2: | fructofuranosidase[e] --> fructofuranosidase [ext] | | |  | |
| for all | R3: | biomass[c] --> biomass [ext] | | | | |
| for all | R4: | NH3 [ext] --> NH3[c] | | | | |
| for all | R5: | SO4 [ext] --> SO4[c] | | | | |
| for all | R6: | O2 [ext] --> O2[e] | | | | |
| for all | R7: | CO2[c] --> CO2[ext] | | | | |
| for all | R8: | ATP_maintenance[c] --> ATP_maintenance[ext] | | | | |
| for all | R9: | oxalate[c] --> oxalate[ext] | | | | |
| S | R10: | soybean oil[ext]--> soybean oil[e] | | | | |
| X | R11: | xylose[ext] --> xylose[e] | | | | |
| g | R12: | glycerol[ext] --> glycerol[e] | | | | |
| for all | R13: | NO3[ext] --> NO3[c] | | | | |
| for all | R14: | gluconate[e] 🡪 gluconate[ext] | | | | |
|  |  |  | | | | |
|  | **Shuttles (mitochondrial, extra-/intra-cellular)** | | |  | | |
| for all | R20: | O2[e] <==> O2[c] | | | | |
| for all | R21: | O2[c] <==> O2[m] | | | | |
| for all | R22: | CO2[c] <==> CO2[m] | | | | |
| for all | R23: | ADP[c] + ATP[m] --> ADP[m] + ATP[c] | | | | |
| for all | R24: | pyruvate[c] <==> pyruvate[m] | | | | |
| for all | R25: | citrate[c] + malate[m] --> citrate[c] + malate[c] | | | | |
| for all | R26: | fumarate[c] <==> fumarate[m] | | | | |
| for all | R27: | isocitrate[c] <==> isocitrate[m] | | | | |
| for all | R28: | succinate[c] <==> succinate[m] | | | | |
| for all | R29: | isocitrate[m] + malate[c] --> isocitrate[c] + malate[m] | | | | |
|  |  |  | | | | |
|  | **Embden-Meyerhof-Parnas Pathway** | | | | |  |
| G | R40: | glucose[e] --> glucose[c] |  | | | |
| for all | R41: | glucose[c] + ATP[c] --> glucose-6-P[c] + ADP[c] | | | | |
| for all | R42: | glucose-6-P[c] <==> fructose-6-P[c] | | | | |
| for all | R43: | fructose-6-P[c] + ATP[c] --> fructose-1,6-bis-P[c] + ADP[c] | | | | |
| for all | R44: | fructose-1,6-bis-P[c] <==> DHAP[c] + GA-3-P[c] | | | | |
| for all | R45: | DHAP[c] <==> GA-3-P[c] | | | | |
| for all | R46: | GA-3-P[c] + ADP[c] + NAD[c] <==> 3-P-glycerate[c] + ATP[c] + NADH[c] | | | | |
| for all | R47: | 3-P-glycerate[c] <==> PEP[c] | | | | |
| for all | R48: | PEP[c] + ADP[c] --> pyruvate[c] + ATP[c] | | | | |
|  |  |  | | | | |
|  | **Fructose and mannose metabolism** | | | | | |
| for all | R53: | fructose[c] + NADPH[c] <==> mannitol[c] + NADP[c] | | | | |
| for all | R54: | fructose-6-P[c] + NADH[c] <==> mannitol-1-P[c] + NAD[c] | | | | |
| for all | R55: | mannose-6-P[c] <==> fructose-6-P[c] | | | | |
| for all | R56: | mannitol-1-P[c] --> mannitol [c] | | | | |
| for all | R57: | fructose[c] + ATP[c] --> fructose-6-P[c] + ADP[c] | | | | |
|  |  |  | | | | |
|  | **Gluconeogenesis** | | | | | |
| for all | R63: | glucose-6-P[c] --> glucose[c] | | | | |
| for all | R64: | fructose-1,6-bis-P[c] --> fructose-6-P[c] | | | | |
| for all | R65: | oxaloacetate[c] + ATP[c] --> PEP[c] + ADP[c] + CO2[c] | | | | |
| for all | R66: | pyruvate[c] + ATP[c] + CO2[c] --> oxaloacetate[c] + ADP[c] | | | | |
|  |  |  | | | | |
|  | **Pentose phosphate pathway** | | | | | |
| G,X | R70: | glucose[e] + O2[e] --> gluconate[e] + H2O2[e] | | | | |
| G,X | R71: | gluconate[e] --> gluconate[c] | | | | |
| G,X | R72: | (2) H2O2[e] --> O2[e] | | | | |
| G,X | R73: | gluconate[c] + ATP[c] --> gluconate-6-P[c] + ADP[c] | | | | |
| for all | R74: | glucose 6-P[c] + NADP[c] --> gluconate-6-P[c] + NADPH[c] | | | | |
| for all | R75: | gluconate 6-P[c] + NADP[c] --> ribulose-5-P[c] + CO2[c] + NADPH[c] | | | | |
| for all | R76: | ribulose 5-P[c] <==> xylulose 5-P[c] | | | | |
| for all | R77: | ribulose 5-P[c] <==> ribose 5-P[c] | | | | |
| for all | R78: | ribose 5-P[c] + xylulose 5-P[c] <==> GA 3-P[c] + sedoheptulose 7-P[c] | | | | |
| for all | R79: | GA-3-P[c] + sedoheptulose 7-P[c] <==> erythrose 4-P[c] + fructose 6-P[c] | | | | |
| for all | R80: | erythrose 4-P[c] + xylulose 5-P[c] <==> fructose 6-P[c] + GA 3-P[c] | | | | |
|  |  |  | | | | |
|  | **Cytosolic reactions** | | | | | |
| for all | R84: | malate[c] <==> fumarate[c] | | | | |
| for all | R85: | malate[c] + NADP[c] --> pyruvate[c] + NADPH[c] + CO2[c] | | | | |
| for all | R86: | NADH[c] + oxaloacetate[c] <==> malate[c] + NAD[c] | | | | |
| for all | R87: | oxaloacetate[c] --> acetate[c] + oxalate[c] | | | | |
| for all | R88: | acetate[c] + ATP[c] --> AcCoA[c] + ADP[c] | | | | |
| for all | R89: | oxalate[c] --> formate[c] + CO2[c] | | | | |
| for all | R90: | formate[c] + NAD[c] --> CO2[c] + NADH[c] | | | | |
| for all | R91: | citrate[c] + ATP[c] --> oxaloacetate[c] + AcCoA[c] + ADP[c] | | | | |
| for all | R92: | citrate[c] <==> isocitrate[c] | | | | |
| for all | R93: | isocitrate[c] + NADP[c] --> α-ketoglutarate[c] + CO2[c] + NADPH[c] | | | | |
| for all | R94: | isocitrate[c] + NAD[c] --> α -ketoglutarate[c] + CO2[c] + NADH[c] | | | | |
| for all | R95: | fumarate[c] + FADH2[m] --> succinate[c] + FAD[m] | | | | |
| for all | R96: | NADH[c] + NADP[c] --> NAD[c] + NADPH[c] | | | | |
| for all | R97: | NADPH[c] + NAD[c] --> NADH[c] + NADP[c] | | | | |
| for all | R98: | ATP[c] --> ADP[c] + ATP_maintenance[c] | | | | |
| X | R99: | xylose[e] --> xylose[c] | | | | |
| X | R100: | xylose[c] + NADPH[c] <==> xylitol[c] + NADP[c] | | | | |
| X | R101: | xylitol[c] + NAD[c] <==> xylulose[c] + NADH[c] | | | | |
| X | R102: | xylulose[c] + ATP[c] --> xylulose-5-P[c] + ADP[c] | | | | |
| g, S | R103: | glycerol[e] --> glycerol[c] | | | | |
| g | R104: | glycerol[c] + NAD[c] --> glycerone + NADH[c] | | | | |
| g | R105: | glycerol 3-P[c] + FAD[m] --> DHAP[c] + FADH2[m] | | | | |
| for all | R106: | NO3[c] + (4) NADPH[c] --> NH3[c] + (4) NADP[c] | | | | |
| g | R107: | glycerol[c] + ATP[c] 🡪 glycerol 3-P[c] + ADP[c] | | | | |
| g | R108: | glycerone[c] + ATP[c] 🡪 DHAP[c] + ADP[c] | | | | |
|  |  |  | | | | |
|  |  |  | | | | |
|  | **Glyoxysomal reactions** | | | | | |
| for all | R130: | isocitrate[c] <==> isocitrate[g] | | | | |
| for all | R131: | isocitrate[g] <==> glyoxalate[g] + succinate[g] | | | | |
| for all | R132: | succinate[g] <==> succinate[c] | | | | |
| for all | R133: | glyoxalate[g] + AcCoA[g] --> malate[g] | | | | |
| for all | R134: | malate[g] <==> malate[c] | | | | |
| for all | R135: | AcCoA[c] <==> AcCoA[g] | | | | |
|  |  |  | | | | |
|  | **Mitochondrial reactions / Energy metabolism** | | | | | |
| for all | R139: | citrate[m] + ATP[m] --> oxaloacetate[m] + AcCoA[m] + ADP[m] | | | | |
| for all | R140: | pyruvate[m] + NAD[m] --> AcCoA[m] + NADH[m] + CO2[m] | | | | |
| for all | R141: | AcCoA[m] + oxaloacetate[m] --> citrate[m] | | | | |
| for all | R142: | citrate[m] <==> isocitrate[m] | | | | |
| for all | R143: | isocitrate[m] + NAD[m] --> α -ketoglutarate[m] + CO2[m] + NADH[m] | | | | |
| for all | R144: | isocitrate[m] + NADP[m] --> α -ketoglutarate[m] + CO2[m] + NADPH[m] | | | | |
| for all | R145: | α -ketoglutarate[m] + NAD[m] + ADP[m] --> succinate[m] + NADH[m] + ATP[m] + CO2[m] | | | | |
| for all | R146: | succinate[m] + Q[m] <==> fumarate[m] + QH2[m] | | | | |
| for all | R147: | fumarate[m] <==> malate[m] | | | | |
| for all | R148: | malate[m] + NAD[m] <==> oxaloacetate[m] + NADH[m] | | | | |
| for all | R149: | (2) NADH2[m] + O2[m] + (5.28) ADP[m] --> (2) NAD[m] + (5.28) ATP[m] | | | | |
| for all | R150: | (2) QH2[m] + O2[m] + (3.28) ADP[m] --> (2) Q[m] + (3.28) ATP[m] | | | | |
| for all | R151: | pyruvate[m] + CO2[m] + ATP[m] --> oxaloacetate[m] + ADP[m] | | | | |
| for all | R152: | NADH[c] + Q[m] --> NAD[c] + QH2[m] | | | | |
|  |  |  | | | | |
|  | **Sulphate assimilation** | | | | | |
| for all | R170: | (4) ATP[c] + (4) NADPH[c] + SO4[c] --> (4) ADP[c] + H2S[c] + (4) NADP[c] | | | | |
|  |  |  | | | | |
|  | **Lipid metabolism / degradation of soybean oil in mitochondria and glyoxysomes** | | | | | |
| S | R180: | soybeanoil[e] --> glycerol[e] + (2.1) linolicacid[e] + (0.9) oleicacid[e] | | | | |
| S | R181: | oleicacid[e] --> oleicacid[c] | | | | |
| S | R182: | linolicacid[e] --> linolicacid[c] | | | | |
| S | R184: | oleicacid[c] + ATP[c] --> oleicacid-CoA[c] + AMP[c] | | | | |
| S | R185: | oleicacid-CoA[c] --> oleicacid-CoA[g] | | | | |
| S | R185: | oleicacid-CoA[c] --> oleicacid-CoA[m] | | | | |
| S | R186: | oleicacid-CoA[m] + (7) FAD[m] + (8) NAD[m] --> (9) AcCoA[m] + (7) FADH2[m] + (8) NADH2[m] | | | | |
| S | R187: | oleicacid-CoA[g] + (7) O2[g] + (8) NAD[g] --> (9) AcCoA[g] + (7) H2O2[g] + (8) NADH[g] | | | | |
| S | R188: | H2O2[g] --> O2[g] | | | | |
| S | R189: | O2[g] <==> O2[c] | | | | |
| S | R190: | linolicacid[c] + ATP[c] --> linolicacid-CoA[c] + AMP[c] | | | | |
| S | R191: | linolicacid-CoA[c] --> linolicacid-CoA[g] | | | | |
| S | R192: | linolicacid-CoA[c] --> linolicacid-CoA[m] | | | | |
| S | R193: | linolicacid-CoA[m] + (6) FAD[m] + (8) NAD[m] + NADPH[m] + O2[m] --> (9) AcCoA[m] + (6) FADH[m]  + (8) NADH2[m] + NADP[m] + H2O2[m] | | | | |
| S | R194: | H2O2[m] --> O2[m] | | | | |
| S | R195: | linolicacid-CoA[g] + (7) O2[g] + (8) NAD[g] + NADPH[g] --> (9) AcCoA[g] + (8) NADH[g]  + NADP[g] + (7) H2O2[g] | | | | |
| S | R196: | NADH[c] + NAD[g] <==> NADH[g] + NAD[c] | | | | |
| S | R197: | NADPH[c] + NADP[g] <==> NADPH[g] + NADP[c] | | | | |
| S | R198: | AMP[c] + ATP[c] --> (2) ADP[c] | | | | |
|  |  |  | | | | |
|  | **Fructofuranosidase synthesis / transport [mol/mmol fructofuranosidase]** | | | | | |
| for all | R210: | (0.067) AcCoA[c] + (0.258) pyruvate[c] + (0.126) 3-P-glycerate[c] + (5.482) ATP[c]  + (1.156) NADPH[c] + (0.761) NH3[c] + (0.030) fructose-6-P[c] + (0.018) glucose-6-P[c]  + (0.065) erythrose-4-P[c] + (0.115) PEP[c] + (0.005) H2S[c] + (0.134) oxaloacetate[c]  + (0.116) α -ketoglutarate[c] + (0.308) mannose-6-P[c] + (0.025) ribose-5-P[c] + (0.305) NAD[c]  --> (1) fructofuranosidase[c] + (0.305) NADH[c] + (0.04) CO2[c] + (5.482) ADP[c] + (1.156) NADP[c] | | | | |
|  |  |  | | | | |
| for all | R211: | fructofuranosidase[c] + ATP[c] --> fructofuranosidase[e] + ADP[c] | | | | |
|  |  |  | | | | |
|  | **Biomass synthesis [mol/1000 g biomass]** | | | | | |
| for all | R220: | (1.03) oxaloacetate[c] + (0.89) 3-P-glycerate[c] + (0.37) ribose-5-P[c] + (61) ATP[c]  + (13.18) NADPH[c] + (7.1) NH3[c] + (2.51) NAD[c] + (3.86) AcCoA[c] + (0.08) FADH2[c]  + (0.08) DHAP[c] + (1.6) glucose 6-P[c] + (0.33) O2[c] + (0.44) fructose-6-P[c] + (0.42) mannose-6-P[c] + (1.91) pyruvate[c] + (1.14) α -ketoglutarate[c] + (0.36) erythrose-4-P[c]  + (0.65) PEP[c] + (0.15) H2S[c] + (0.213) mannitol[c] --> biomass[c] + (0.08) FAD[c] + (13.18) NADP[c] + (2.51) NADH[c] + (61) ADP[c] + (0.00281) CO2[c] | | | | |

**Statistical evaluation**

**Table A2: Statistical analysis of simulation data. Growth associated fructofuranosidase production using glucose. R²: regression coefficient, alpha: slope-correlation coefficient, NOSTAT: no statistical evaluation. The values correspond to Figure 7 Glu(+). The entries of ‘#DIV/0’ regarded to constant values (or complete zeros) of stoichiometric coefficients for the corresponding enzyme.**

**Table A3: Statistical analysis of simulation data. Non-growth associated fructofuranosidase production using glucose. R²: regression coefficient, alpha: slope-correlation coefficient, NOSTAT: no statistical evaluation. The values correspond to Figure 7 Glu(-). The entries of ‘#DIV/0’ regarded to constant values (or complete zeros) of stoichiometric coefficients for the corresponding enzyme.**

**Table A4: Statistical analysis of simulation data. Growth associated fructofuranosidase production using xylose. R²: regression coefficient, alpha: slope-correlation coefficient, NOSTAT: no statistical evaluation. The values correspond to Figure 7 Xyl(+). The entries of ‘#DIV/0’ regarded to constant values (or complete zeros) of stoichiometric coefficients for the corresponding enzyme.**

**Table A5: Statistical analysis of simulation data. Non-growth associated fructofuranosidase production using xylose. R²: regression coefficient, alpha: slope-correlation coefficient, NOSTAT: no statistical evaluation. The values correspond to Figure 7 Xyl(-). The entries of ‘#DIV/0’ regarded to constant values (or complete zeros) of stoichiometric coefficients for the corresponding enzyme.**

**Table A6: Statistical analysis of simulation data. Growth associated fructofuranosidase production using glycerol. R²: regression coefficient, alpha: slope-correlation coefficient, NOSTAT: no statistical evaluation. The values correspond to Figure 7 Gly(+). The entries of ‘#DIV/0’ regarded to constant values (or complete zeros) of stoichiometric coefficients for the corresponding enzyme.**

**Table A7: Statistical analysis of simulation data. Non-growth associated fructofuranosidase production using glycerol. R²: regression coefficient, alpha: slope-correlation coefficient, NOSTAT: no statistical evaluation. The values correspond to Figure 7 Gly(-). The entries of ‘#DIV/0’ regarded to constant values (or complete zeros) of stoichiometric coefficients for the corresponding enzyme.**

**Table A8: Statistical analysis of simulation data. Growth associated fructofuranosidase production using oleic acid. R²: regression coefficient, alpha: slope-correlation coefficient, NOSTAT: no statistical evaluation. The values correspond to Figure 7 Oel(+). The entries of ‘#DIV/0’ regarded to constant values (or complete zeros) of stoichiometric coefficients for the corresponding enzyme.**

**Table A9: Statistical analysis of simulation data. Growth associated fructofuranosidase production using oleic acid. R²: regression coefficient, alpha: slope-correlation coefficient, NOSTAT: no statistical evaluation. The values correspond to Figure 7 Oel(-). The entries of ‘#DIV/0’ regarded to constant values (or complete zeros) of stoichiometric coefficients for the corresponding enzyme.**

**Reference**

1. Andersen MR, Nielsen ML, Nielsen J: **Metabolic model integration of the bibliome, genome, metabolome and reactome of Aspergillus niger**. *Mol Syst Biol* 2008, **4**:178.

2. David H, Åkesson M, Nielsen J: **Reconstruction of the central carbon metabolism of *Aspergillus niger***. *European Journal of Biochemistry* 2003, **270**(21):4243-4253.

3. Diano A, Bekker-Jensen S, Dynesen J, Nielsen J: **Polyol synthesis in *Aspergillus niger*: Influence of oxygen availability, carbon and nitrogen sources on the metabolism**. *Biotechnology and Bioengineering* 2006, **94**(5):899-908.

4. Pel HJ, de Winde JH, Archer DB, Dyer PS, Hofmann G, Schaap PJ, Turner G, de Vries RP, Albang R, Albermann K *et al*: **Genome sequencing and analysis of the versatile cell factory *Aspergillus niger* CBS 513.88**. *Nat Biotechnol* 2007, **25**(2):221-231.

5. van Kuyk PA, Diderich JA, MacCabe AP, Hererro O, Ruijter GJ, Visser J: ***Aspergillus niger mstA* encodes a high-affinity sugar/H+ symporter which is regulated in response to extracellular pH**. *Biochem J* 2004, **379**(Pt 2):375-383.

6. Jernejc K, Legisa M: **Purification and properties of carnitine acetyltransferase from citric acid producing *Aspergillus niger***. *Appl Biochem Biotechnol* 1996, **60**(2):151-158.

7. Steinbock F, Choojun S, Held I, Roehr M, Kubicek CP: **Characterization and regulatory properties of a single hexokinase from the citric acid accumulating fungus *Aspergillus niger***. *Biochim Biophys Acta* 1994, **1200**(2):215-223.

8. Prathumpai W, Gabelgaard JB, Wanchanthuek P, van de Vondervoort PJ, de Groot MJ, McIntyre M, Nielsen J: **Metabolic control analysis of xylose catabolism in *Aspergillus***. *Biotechnol Prog* 2003, **19**(4):1136-1141.

9. Vasilev N, Vasileva M, Ganchev I: **Changes in the activity of Krebs cycle enzymes in an *Aspergillus niger* strain during the biosynthesis of citric acid**. *Acta Microbiol Bulg* 1985, **17**:46-51.

10. Osmani SA, Scrutton MC: **The sub-cellular localisation of pyruvate carboxylase and of some other enzymes in *Aspergillus nidulans***. *Eur J Biochem* 1983, **133**(3):551-560.

11. Bercovitz A, Peleg Y, Battat E, Rokem JS, Goldberg I: **Localization of pyruvate carboxylase in organic acid-producing *Aspergillus* strains**. *Appl Environ Microbiol* 1990, **56**(6):1594-1597.

12. Alvarez-Vasquez F, Gonzalez-Alcon C, Torres NV: **Metabolism of citric acid production by *Aspergillus niger*: model definition, steady-state analysis and constrained optimization of citric acid production rate**. *Biotechnol Bioeng* 2000, **70**(1):82-108.

13. Kubicek CP, Schreferl-Kunar G, Wohrer W, Rohr M: **Evidence for a cytoplasmic pathway of oxalate biosynthesis in *Aspergillus niger***. *Appl Environ Microbiol* 1988, **54**(3):633-637.

14. Hult K, Veide A, Gatenbeck S: **The distribution of the NADPH regenerating mannitol cycle among fungal species**. *Arch Microbiol* 1980, **128**(2):253-255.

15. Ruijter GJ, Bax M, Patel H, Flitter SJ, van de Vondervoort PJ, de Vries RP, vanKuyk PA, Visser J: **Mannitol is required for stress tolerance in *Aspergillus niger* conidiospores**. *Eukaryot Cell* 2003, **2**(4):690-698.

16. Falony G, Armas JC, Dustet Mendoza JC, Martinez Hernandez JL: **Production of extracellular lipase from *Aspergillus niger* by solid state fermentation**. *Food Technology and Biotechnology* 2006, **44**(2):235-240.

17. Maggio-Hall LA, Keller NP: **Mitochondrial beta-oxidation in *Aspergillus nidulans***. *Mol Microbiol* 2004, **54**(5):1173-1185.
